# Schumann-anchored golden ratio organization of human neural oscillations

**DOI:** 10.3389/fncom.2026.1786996

**Published:** 2026-06-02

**Authors:** Michael Lacy

**Affiliations:** Independent Researcher, Austin, TX, United States

**Keywords:** EEG, FOOOF, golden ratio, neural oscillations, Schumann resonance, spectral parameterization, EEG band definitions, cross-frequency coupling

## Abstract

**Introduction:**

Human neural oscillations are organized according to golden ratio (φ = 1.618) mathematics: frequencies follow $f\left(n\right)=f_{0}\times \varphi^{n}$ where *f*_0_≈7.6 Hz. This architecture manifests as spectral peak depletion at integer *n* positions (band boundaries) and enrichment at half-integer positions (band centers), providing empirical validation of a previously theorized φ^*n*^ architecture and identifying the absolute fundamental frequency *f*_0_ = 7.6 Hz. This organization was discovered and validated through two complementary studies.

**Study 1—transient events:**

Analysis of 1,366 Schumann Ignition Events (SIEs)—transient episodes of multi-band network synchronization at Earth-resonant frequencies—across 91 participants, 661 sessions, and three EEG devices characterized harmonic frequencies that suggested φ^*n*^ relationships (< 1% mean ratio error). Individual frequencies varied independently across events (all |*r*| < 0.03), yet ratio precision was preserved—an “independence-convergence paradox” indicating population-level rather than event-level constraints. Null controls confirmed genuine organization (Cohen's *d* = 1.44, *p* < 0.0001).

**Study 2—single-channel spectral architecture:**

Spectral parameterization of 244,955 oscillatory peaks across 968 sessions confirmed predictions derived from the φ^*n*^ framework: boundaries showed −18% depletion, attractors +21% enrichment, and noble positions (*n*+0.618) +39% enrichment in aggregate cross-band analysis. The framework extends to an eight-position hierarchy including “inverse nobles” (*n*+0.764, *n*+0.854)—symmetric to regular nobles about the attractor—which inherit stability through multi-scale Fibonacci pathways. Gamma exhibited strongest aggregate adherence (+144.8% at Noble1 in cross-band analysis), consistent with functional requirements for precise phase relationships, though this aggregate figure may partially reflect cross-band density effects (see Section 6.8, Limitation 5). Independent replication in the EEGEmotions-27 dataset (612,990 peaks, 2,342 sessions) confirmed the same qualitative pattern with Kendall's τ = 1.0.

**Synthesis:**

Two independent methodological approaches—transient event detection and single-channel spectral parameterization—converge on identical conclusions: neural oscillations follow φ^*n*^ organization with perfect position ordering (Kendall's τ = 1.0) across all analyses. The fundamental frequency *f*_0_ = 7.6 Hz emerges independently from geophysical monitoring of Earth's Schumann Resonance and from neural spectral optimization, agreeing within 0.4%. These findings support a “substrate-ignition” model: the φ^*n*^ lattice exists continuously as an architectural scaffold organizing neural oscillations, while transient high-coherence events (SIEs) represent moments when this substrate is amplified and frequencies “snap” into tighter compliance. The golden ratio's unique mathematical properties—maximal resistance to mode-locking between frequency bands combined with precise Fibonacci-mediated cross-frequency coupling pathways—may represent evolution's solution to a fundamental computational challenge: maintaining independent parallel processing streams (segregation) while enabling flexible, controlled communication between them (integration).

## 1 Introduction

### 1.1 Transient synchronization phenomena in EEG

Neural oscillations are fundamental to brain function, coordinating activity across distributed networks to support perception, cognition, and behavior (Buzsáki, 2006; Fries, 2015). These rhythmic fluctuations in neuronal population activity occur across characteristic frequency bands—from slow delta oscillations (< 4 Hz) through high gamma (>80 Hz)—each associated with distinct functional roles (Klimesch, 1999; Engel and Fries, 2010; Cohen, 2017). Although substantial research has characterized sustained oscillatory states and their behavioral correlates, transient synchronization phenomena—brief episodes of enhanced coordination across brain regions—have received comparatively less systematic investigation despite their potential significance for understanding rapid neural state transitions and information integration (Siegel et al., 2012; Canolty and Knight, 2010).

Well-characterized examples of transient oscillatory events include sleep spindles (Fernandez and Lüthi, 2020), hippocampal sharp-wave ripples (Buzsáki, 2015), and task-related gamma bursts (Fries et al., 2001). These phenomena share common features: discrete temporal boundaries, elevated power relative to baseline, and enhanced inter-regional synchronization. Such transient events may carry functional significance distinct from sustained oscillations, potentially serving as temporal windows for memory consolidation, inter-regional communication, or state transitions (Lisman and Buzsáki, 2008).

The overlap between Earth's Schumann Resonance frequencies (fundamental ~7.83 Hz with harmonics at ~14.3, 20.8, 27.3, and 33.8 Hz) and canonical EEG bands has prompted investigation into potential brain-environment relationships (König et al., 1981; Cherry, 2002; Persinger, 2008; Saroka et al., 2016). However, prior studies have mainly employed correlational designs examining continuous SR-EEG relationships rather than systematically detecting and characterizing discrete events. The temporal structure, duration, and network dynamics of individual SR-frequency episodes have remained poorly characterized.

During exploratory analysis of EEG recordings from meditation sessions collected between 2019 and 2024, I repeatedly observed transient periods of elevated power spectral density at approximately 7.6–7.8 Hz across multiple brain regions. These events involved coordinated power increases at multiple frequencies, accompanied by marked elevation in inter-channel coherence and phase synchronization—suggesting network-wide coordination rather than isolated oscillatory bursts. This discovery prompted systematic investigation, revealing an unexpected mathematical structure in the frequency relationships that forms the basis of the present paper.

### 1.2 The problem of EEG frequency band definitions

Since Hans Berger's discovery of the human alpha rhythm in 1929, electroencephalography has been organized around a taxonomy of frequency bands: delta (< 4 Hz), theta (4–8 Hz), alpha (8–13 Hz), beta (13–30 Hz), and gamma (>30 Hz). These bands emerged from empirical observation and clinical utility rather than theoretical derivation.

A survey of band definitions across published EEG studies (Table 1) reveals substantial inconsistency:

**Table 1:** Variability in EEG Band Definitions Across Published Literature.

Band	Lower bound range	Upper bound range	Variation
Delta	0.1–1.0 Hz	3.0–4.0 Hz	1.0 Hz
Theta	3.0–5.0 Hz	7.0–8.0 Hz	3.0 Hz
Alpha	7.0–9.0 Hz	12.0–14.0 Hz	4.0 Hz
Beta	12.0–15.0 Hz	25.0–35.0 Hz	13.0 Hz
Gamma	25.0–40.0 Hz	80.0–150.0 Hz	85.0 Hz

Band definition ranges compiled by the author from representative published studies spanning 1999–2024, including Klimesch (1999), Buzsáki (2006), Cohen (2017), and Herrmann et al. (2016).

This variability suggests that current band definitions may not reflect natural categories in neural organization. If frequency bands represented genuine biological discontinuities, convergence on boundary positions would be expected. The observed divergence implies either (a) bands are arbitrary conveniences, or (b) the true boundaries exist but have not been identified.

Evidence for the latter comes from Pletzer et al. (2010), who observed that adjacent EEG frequency bands form a geometric series with ratio approximating the golden mean (φ≈1.618): delta2/delta1 ≈ 1.67, beta1/alpha = 1.60, gamma1/beta2 = 1.60. They proved mathematically that this architecture provides maximal desynchronization between bands during rest while enabling controlled coupling during active processing. Recent independent work by Kramer (2022) provides complementary theoretical support, proving that φ is the *unique* multiplicative factor enabling cross-frequency coupling where all participating frequencies belong to the same geometric series. However, prior frameworks used individual alpha frequency (~10 Hz) as a relative anchor rather than identifying an absolute fundamental frequency. The present work extends this theoretical foundation by discovering the fundamental frequency *f*_0_ = 7.6 Hz and validating the φ^*n*^ architecture empirically across 244,955 spectral peaks.

As will be demonstrated in this paper, the discovery of golden ratio frequency ratios in transient high-coherence events provides a principled mathematical framework that may resolve this long-standing ambiguity.

### 1.3 Schumann resonance as potential reference

The Earth's ionospheric cavity supports a set of global electromagnetic resonances, termed Schumann Resonances (SR), arising from electromagnetic waves circling the Earth-ionosphere waveguide driven primarily by global lightning activity (Schumann, 1952; Nickolaenko and Hayakawa, 2014). For a simplified cavity model, the fundamental frequency is given by Equation 1:


$$\begin{array}{l} f_{1}^{approx}=\frac{c}{2\pi r}=\frac{299,792,458}{2\pi \times 6,371,000}=7.49\text{Hz} \end{array}$$ (1)

where *c* is the speed of light and *r* is Earth's radius.

Long-term geophysical monitoring provides direct measurement of SR parameters. The Tomsk Space Observing System (Russia), operating continuously since 1994, reports the fundamental centered at 7.6 ± 0.2 Hz with diurnal and seasonal variations of ±0.3 Hz. The “canonical” value of 7.83 Hz frequently cited in literature represents a theoretical approximation; empirical monitoring consistently yields lower values near 7.6 Hz.

**Critical Methodological Point**

Before proceeding, it should be emphasized that correspondence between neural and Schumann frequencies need not imply direct electromagnetic coupling. Three explanations are possible:

**(1) Evolutionary tuning:** Schumann Resonances have existed for ~4 billion years (since Earth developed an ionosphere), providing ample evolutionary time. If SR frequencies offer an optimal “temporal scaffold” for neural coordination, organisms whose oscillations aligned with this environmental constant may have gained selective advantages: improved internal synchronization, enhanced inter-organism coordination in social species, and access to a stable external reference clock.

**(2) Biophysical convergence:** Independent optimization to similar frequencies due to shared physical constraints (characteristic length scales, resonance conditions in conductive media).

**(3) Direct coupling:** Real-time electromagnetic interaction between brain and ionosphere (least supported given ~picoTesla field strengths).

These explanations are not mutually exclusive—evolutionary tuning *to* biophysically optimal frequencies is plausible. The present study documents the φ^*n*^ architecture without requiring resolution of this question, though Section 6.4 discusses the evidence for each hypothesis.

The proximity of the SR fundamental (~7.6 Hz) to the theta-alpha boundary in human EEG has been noted in previous work. However, investigations have been limited by lack of a theoretical framework specifying *what pattern* to expect if neural frequencies were organized relative to SR. The present study arose from the unexpected discovery of precise mathematical relationships in the harmonic structure of transient neural events—relationships that suggested a framework based on the golden ratio rather than simple integer harmonics.

### 1.4 Study overview: from discovery to validation

This paper follows the actual trajectory of discovery:

Phase 1 (Discovery): During analysis of meditation EEG recordings, transient high-coherence events showing multi-band synchronization at specific frequencies falling within the Schumann Resonance range were detected. These were termed “Schumann Ignition Events” (SIEs). Initial characterization revealed 1,366 events across 91 subjects and five cognitive contexts, with robust network synchronization signatures.

Phase 2 (Pattern Recognition): Upon examining the harmonic frequencies detected in SIE events—particularly the relationships between SR1 (~7.6 Hz), SR3 (~20 Hz), and SR5 (~32 Hz)—it was observed that their ratios approximated powers of the golden ratio (φ = 1.618). This observation, while potentially confounded by the SR-derived search windows (which by design fall near φ^*n*^ lattice positions), motivated systematic investigation through unconstrained methods.

Phase 3 (Hypothesis Generation): The discovery of φ^*n*^ ratios in transient events raised a fundamental question: Is this organization specific to high-coherence states, or does it reflect a deeper architectural principle governing all EEG frequency organization? The hypothesis was formalized as $f\left(n\right)=f_{0}\times \varphi^{n}$ and specific predictions for how spectral peaks should be distributed were derived.

Phase 4 (Validation): Systematic analysis of 244,955 oscillatory peaks across 968 EEG sessions confirmed the predicted boundary-attractor structure: depletion at integer φ^*n*^ positions, enrichment at half-integer positions, and maximal enrichment at “noble” positions (*n*+0.618).

The following sections present these phases in sequence, beginning with the full methodological and empirical characterization of SIE discovery (Section 2), followed by the theoretical framework that emerged from that discovery (Section 3), single-channel spectral validation testing predictions in continuous EEG (Section 4), and the integration of both lines of evidence (Section 5).

The present study addresses two primary research questions: (1) Are the harmonic frequencies observed during transient high-coherence events organized according to golden ratio (φ = 1.618) scaling? (2) Does this φ-ratio organization extend beyond transient events to govern the continuous spectral architecture of EEG? Based on the mathematical properties of the golden ratio—maximal anti-commensurability and Fibonacci additivity—the hypothesis is that EEG frequency organization follows $f\left(n\right)=f_{0}\times \varphi^{n}$, generating specific predictions for where spectral peaks should cluster (attractor and noble positions) and where they should be depleted (boundary positions).

## 2 Study 1: discovery and characterization of Schumann ignition events

### 2.1 Methods: SIE detection and analysis

#### 2.1.1 Overview

This study employed a two-phase design: (1) discovery and initial characterization of Schumann Ignition Events (SIEs) in longitudinal meditation EEG recordings, and (2) validation and extension in independent datasets collected during cognitive tasks. The initial discovery and characterization constitutes an exploratory, hypothesis-generating analysis. The core discovery originated from longitudinal recordings of a single experienced meditator, with subsequent validation extending to 91 participants across five cognitive contexts. All analyses were performed in Python 3.9+ using NumPy (NumFOCUS Foundation, Austin, TX, USA), SciPy (NumFOCUS Foundation, Austin, TX, USA), MNE-Python (Athinoula A. Martinos Center for Biomedical Imaging, Massachusetts General Hospital, Boston, MA, USA) (Harris et al., 2020; Virtanen et al., 2020; Gramfort et al., 2013), and the FOOOF library (Voytek Lab, Department of Cognitive Science, University of California San Diego, La Jolla, CA, USA) (Donoghue et al., 2020).

Terminology note: The term “Schumann Ignition Events” reflects the initial discovery of transient ignitions—brief episodes of elevated amplitude, coherence, and phase-locking at frequencies near Earth's Schumann Resonance fundamental (~7.8 Hz). The name references these observed characteristics rather than demonstrated electromagnetic coupling between neural and geophysical systems. Whether the frequency correspondence reflects evolutionary tuning, biophysical convergence, or coincidence remains an open empirical question requiring concurrent neural and geomagnetic recording.

#### 2.1.2 Participants and recordings

Discovery Dataset: Longitudinal Meditation Recordings. The initial discovery and characterization of SIEs was conducted using EEG recordings from a single experienced meditation practitioner (male, age range 44–48 years, >10 years meditation experience). Recordings were collected during eyes-closed meditation sessions spanning November 2019 to March 2024, comprising 34 sessions with a total recording duration of approximately 7.5 h. Three consumer-grade EEG devices were used across sessions to enable cross-device validation:

- Muse (2016 model; InteraXon Inc., Toronto, Ontario, Canada) (Muse devices): 4 channels (TP9, AF7, AF8, TP10; dry electrodes), 256 Hz sampling rate, FPz reference, 17 sessions
- Emotiv EPOC X (Emotiv Inc., San Francisco, CA, USA) (EPOC X, Insight): 14 channels (AF3, F7, F3, FC5, T7, P7, O1, O2, P8, T8, FC6, F4, F8, AF4; saline-soaked felt pads), 128 Hz sampling rate, CMS/DRL reference, 5 sessions.
- Emotiv Insight (Emotiv Inc., San Francisco, CA, USA) (EPOC X, Insight): 5 channels (AF3, AF4, T7, T8, Pz; semi-dry polymer sensors), 128 Hz sampling rate, CMS/DRL reference, 12 sessions.

Validation Datasets. To test generalization beyond meditation, three publicly available datasets were analyzed:

- PhySF (Irshad et al., 2023): *N* = 25 participants performing mathematical tasks designed to induce flow states, recorded with Emotiv EPOC X.
- MultiPENG (Rashed et al., 2025): *N* = 36 participants playing video games across varying difficulty levels (Kaggle: 10.34740/kaggle/ds/6552328).
- VEP (Tiwari et al., 2025): *N* = 29 participants viewing natural object images (Emotiv EPOC X) (Mendeley Data: 10.17632/g9shp2gxhy.2).

#### 2.1.3 EEG pre-processing

Raw EEG signals were high-pass filtered at 1 Hz using a 4th-order zero-phase Butterworth filter (Butterworth, 1930) to remove slow drifts while preserving oscillatory activity in the Schumann Resonance frequency range. All electrodes were positioned according to the international 10–20 system (Jasper, 1958).

#### 2.1.4 Schumann resonance harmonic detection

Canonical Frequencies. Canonical frequencies and search bandwidths were derived from long-term magnetometer data recorded by the Space Observing System in Tomsk, Russia: SR1 = 7.6 Hz (±0.6 Hz), SR2o = 13.75 Hz (±0.75 Hz), SR3 = 20 Hz (±1.0 Hz), SR4 = 25 Hz (±2.0 Hz), and SR5 = 32 Hz (±2.5 Hz).

Methodological Note on Search Windows. The search windows were derived from Tomsk magnetometer observations of Earth's Schumann Resonances. Critically, the empirical SR frequencies (7.6, 20, 32 Hz) happen to align closely with φ^*n*^ predictions (φ^0^, φ^2^, φ^3^ positions)—a correspondence that motivated this investigation but also means Study 1 constitutes a *constrained* test of φ^*n*^ organization.

Limitations of within-window precision analysis: By design, 100% of detected harmonic frequencies fall within these predefined windows. A naive null model (uniform random sampling within windows) yields expected φ-error of 4.75%, compared to observed 0.64%. However, this comparison is methodologically limited: (1) FOOOF is a peak-finding algorithm that locates the strongest spectral peak within each window, not a uniform sampler; (2) inherent EEG spectral structure—including alpha (~10 Hz), beta (~20 Hz), and gamma peaks—would produce consistent peak locations regardless of φ^*n*^ architecture. Consequently, Study 1 *characterizes* SIE phenomenology and harmonic relationships, but cannot independently validate φ^*n*^ organization. Study 2's agnostic spectral analysis (Section 4), which detects peaks without reference to predefined windows, provides the unconstrained validation test.

Spectral Parameterization Using FOOOF. To separate oscillatory (periodic) activity from aperiodic (1/f) background noise, FOOOF spectral parameterization was employed (Donoghue et al., 2020), modeling the power spectrum via Equation 2:


$$\begin{array}{l} \log_{10}\left(PSD\left(f\right)\right)=L\left(f\right)+\underset{i}{\sum }G_{i}\left(f\right) \end{array}$$ (2)

where *L*(*f*) = *b*−χ × log_10_(*f*) represents the aperiodic component and *G*_*i*_(*f*) represents Gaussian periodic peaks. FOOOF parameters: frequency range 1–50 Hz, maximum 20 peaks, minimum peak height 0.0001 log units, peak width limits 0.2–20.0 Hz.

#### 2.1.5 Seven-stage detection pipeline

A seven-stage pipeline was developed to systematically identify, refine, and validate candidate SIE events:

Stage 1: Envelope Thresholding. A composite SR envelope was computed by averaging all EEG channels, bandpass filtering at the fundamental frequency (*f*_0_±0.6 Hz), computing the Hilbert envelope, and z-score normalizing. Candidate onsets were identified where *z*(*t*)≥3.0. The z-score threshold of 3.0 (3 SD above session mean) identifies events with unambiguous power elevation while maintaining a detection rate of approximately 2 events per session. At *R* = 0.6, the probability of chance phase alignment across *N*≥4 independent channels is < 0.01 by circular statistics.

Stage 2: FOOOF Harmonic Refinement. For each candidate window, FOOOF was applied to extract session-specific harmonic frequencies within SR search windows.

Stage 3: Kuramoto Phase Synchronization. The precise ignition onset *t*_0_ was refined using the Kuramoto order parameter (Equation 3):


$$R(t)=\left|\frac{1}{N}\sum_{k=1}^{N}e^{i\phi_{k}(t)}\right|$$ (3)

where ϕ_*k*_(*t*) is the instantaneous phase of channel *k* at SR1 frequency. Ignition onset was defined as the time of maximum *dR*/*dt* where *R*(*t*)≥0.6.

Stage 4: Coherence Characterization. Magnitude-squared coherence (MSC) and phase-locking value (PLV) (Lachaux et al., 1999) were computed for each harmonic, with PLV given by Equation 4:


$$PLV=\frac{1}{T}\left|\sum_{t=1}^{T}e^{i\text{Δ}\phi_{xy}(t)}\right|$$ (4)

Stage 5: Harmonic Organization. The Harmonic Stack Index (HSI) quantified the ratio of overtone power to fundamental power: *HSI* = *P*_overtones_/*P*_*f*_.

Stage 6: Quality Metrics. Spectral slope (β) and Frequency Specificity Index (FSI = *P*_*SR*_/*P*_*total*_) were computed.

Stage 7: Composite Scoring. A composite SR-Score was computed using the three observed Schumann harmonics (SR1, SR3, SR5) as defined in Equation 5:


$$\text{SR-Score}=\frac{{\text{Z}}_{\text{weighted}}^{\text{0}.\text{7}}\times {\text{MSC}}_{\text{weighted}}^{\text{1}.\text{2}}\times {\text{PLV}}_{\text{weighted}}}{\text{1+HSI}}$$ (5)

with φ-derived weights: SR1 (0.618), SR3 (0.326), SR5 (0.146), giving greatest emphasis to the fundamental frequency where synchronization is strongest and most reliable.

#### 2.1.6 Null control analyses

Six null control analyses validated that SIE characteristics represent genuine neural organization:

Null Control A (Phase Randomization): For each SIE, phases were randomized while preserving amplitude spectra (100 surrogates per event), testing whether synchronization reflects genuine phase coupling.

Null Control B (Random Temporal Windows): 1,366 random 3-s baseline windows were sampled from periods >10 s from any SIE, testing whether SR organization is event-specific.

Null Control C (Uniform Random Triplets) 10,000 random frequency triplets sampled uniformly from physiologically plausible ranges tested whether φ precision arises by chance.

Null Control D (Peak-Based Random Triplets): 10,000 triplets sampled from actual FOOOF peaks (*N* = 244,955 peaks across 968 sessions) provided a more stringent test controlling for EEG spectral structure.

Null Control E (Shuffled Bootstrap): SR1, SR3, SR5 frequencies were independently permuted across events (1,000 iterations), testing whether ratio precision requires within-event coordination. Results: composite error *p* = 0.030 (marginally significant); individual ratios SR3/SR1, SR5/SR1, SR5/SR3 all *p*>0.05 (non-significant). Interpretation: observed ratio precision is consistent with marginal distributional properties rather than event-level frequency coordination.

Null Control F (Distributional Null Model): Synthetic datasets generated from observed frequency distributions tested population-level encoding.

#### 2.1.7 Statistical power considerations

As this study combines exploratory discovery with confirmatory validation, sensitivity power analysis was employed to characterize the minimum detectable effect sizes given the sample sizes. All power calculations assumed α = 0.05 (two-tailed) and target power of 0.80.

Study 1 (SIE Detection): With *N* = 1, 121 valid harmonic triplets, there was 80% power to detect:

- Cohen's *d*≥0.084 for one-sample t-tests (ratio deviation from φ^*n*^).
- Pearson |*r*|≥0.084 for correlation tests (frequency independence).
- Cohen's *d*≥0.088 for two-sample comparisons (peak-based null control).

Study 2 (Spectral Validation): With *N* = 244, 955 peaks, sensitivity analysis indicated effectively unlimited power for position enrichment comparisons. For the more conservative session-level analysis (*N* = 968), there was 80% power to detect Cohen's *d*≥0.09.

ANOVA analyses: For device independence (3 groups, *N*≈370 per group on average), there was 80% power to detect Cohen's *f* = 0.16 (small-to-medium effect). For context independence (5 groups, unequal sizes ranging from 55 to 604), power exceeded 80% for *f*≥0.125.

### 2.2 Results: SIE characteristics and harmonic structure

#### 2.2.1 Dataset overview and event detection

Application of the seven-stage detection pipeline yielded 1,366 discrete events across 661 recording sessions from 91 unique participants (Table 2). Events were detected across all six datasets and five cognitive contexts, with overall mean detection rate of 2.1 ± 3.3 events per session (range: 1–27). Event duration averaged 26.9 ± 21.3 s (median: 20.0 s), consistent with the transient nature of SIE phenomena and characteristic timescales of transient oscillatory events (Siegel et al., 2012).

**Table 2:** Combined dataset characteristics.

Dataset	Participants	Sessions	SIE Events	Device(s)	Context
Meditation (Files)	1	5	38	EPOC X	Eyes-closed meditation
Meditation (Muse)	1	17	165	Muse	Eyes-closed meditation
Meditation (Insight)	1	12	57	Insight	Eyes-closed meditation
PhySF	25	46	447	EPOC X	Cognitive flow/non-flow tasks
MultiPENG	36	533	604	EPOC X	Video game engagement
VEP	29	48	55	EPOC X	Passive visual perception
**Combined**	**91**	**661**	**1,366**	**3 types**	**5 contexts**

Bold values indicate aggregate totals across all subgroups.

PhySF cognitive flow recordings and meditation recordings yielded the highest per-session detection rates (9.7 and 7.6 events/session respectively), with gaming and visual perception tasks showing lower rates (~1 event/session). This pattern may reflect differential engagement of SR-aligned neural mechanisms across cognitive states. Complete per-event metadata for all 1,366 SIEs, including frequency, coherence, phase-locking, and quality metrics, are provided in Supplementary Data Sheet 1.

#### 2.2.2 Harmonic frequency definitions

Spectral analysis of detected SIEs revealed nine distinct harmonic frequencies spanning the 7–41 Hz range (Table 3). The fundamental frequency (SR1) centers near 7.6 Hz at the theta/alpha boundary, with higher harmonics extending through alpha, beta, and gamma bands.

**Table 3:** φ^*n*^ model predictions vs. measured harmonic frequencies.

Harmonic	*n*	φ^*n*^	Predicted (Hz)	Measured (Hz)	Error (%)	*N*	EEG band
SR1	0	1.000	7.60	7.63 ± 0.33	+0.4	1121	θ/α Boundary
SR1.5	0.5	1.272	9.67	9.96 ± 0.35	+3.0	1139	α Attractor
SR2	1	1.618	12.30	11.98 ± 0.40	−2.6	1050	α/β Boundary
SR2o^*^	–	–	(14.3)	13.76 ± 0.44	–	1150	Low β
SR2.5	1.5	2.058	15.64	15.50 ± 0.46	−0.9	1188	β Attractor
SR3	2	2.618	19.90	19.99 ± 0.57	+0.5	1250	β Boundary
SR4	2.5	3.330	25.31	24.98 ± 1.17	−1.3	1359	High β Attractor
SR5	3	4.236	32.19	32.57 ± 1.23	+1.2	1364	β/γ Boundary
SR6	3.5	5.388	40.95	40.11 ± 1.61	−2.1	1365	γ Attractor

Predictions use *f*(*n*) = 7.6 × φ^*n*^. *N* = events with detectable harmonic. Mean absolute error across φ^*n*^ harmonics: 1.5%.

^*^SR2o corresponds to the canonical 2nd Schumann Resonance harmonic (~14.3 Hz), which does not align with the φ^*n*^ lattice. See Section 6.4.1 for discussion of theoretical implications.

#### 2.2.3 Synchronization metrics

**Figure 1:**
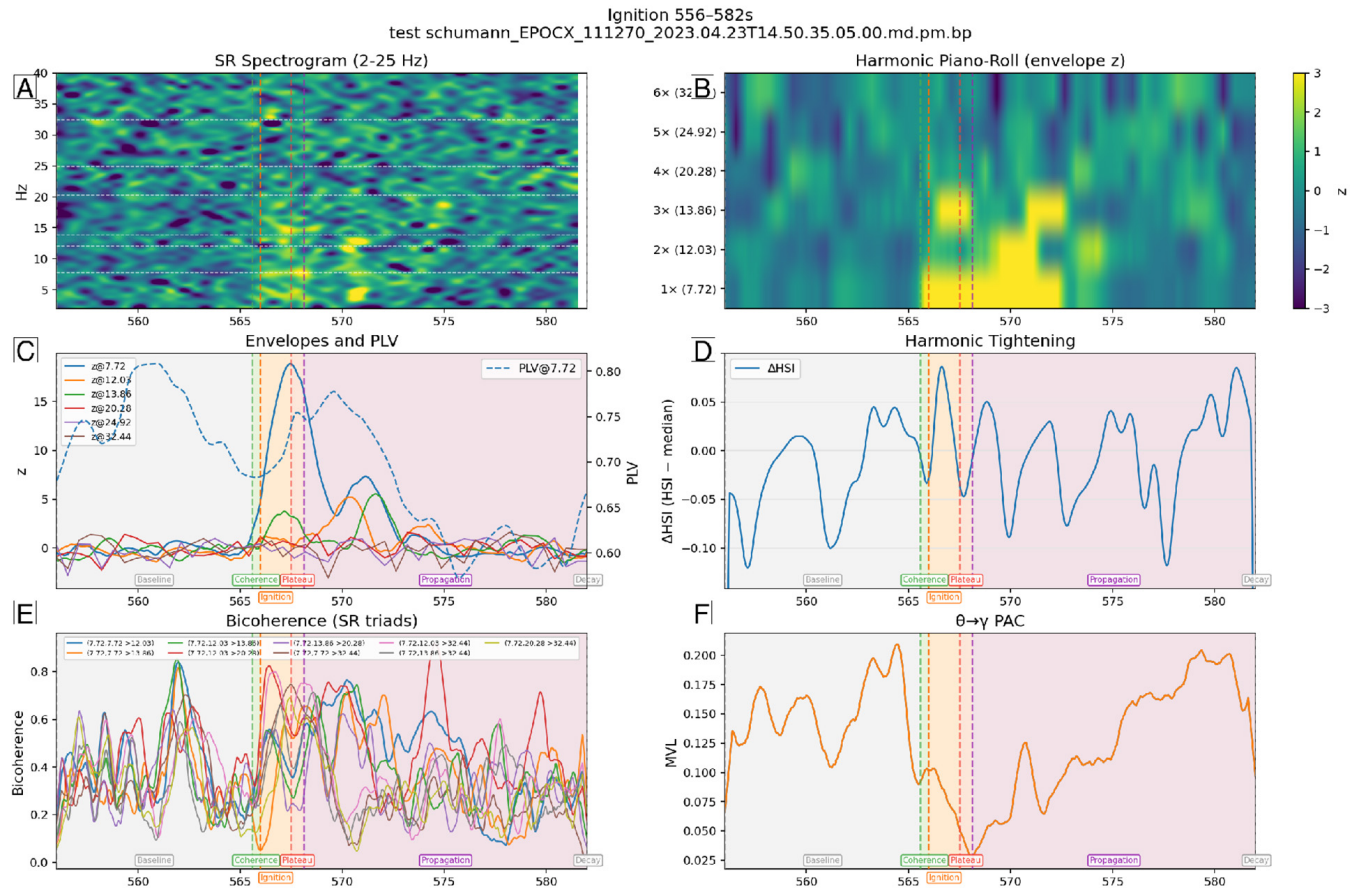
Exemplar SIE event spectral and temporal dynamics. **(A)** Spectrogram (2–25 Hz) showing harmonic activation at φ^*n*^ frequencies. **(B)** Harmonic piano-roll showing z-score elevation across all six harmonics. **(C)** Envelope traces overlaid with phase-locking value, demonstrating coherence-first dynamics. **(D)** Harmonic tightening index showing fundamental dominance preceding ignition. **(E)** Bicoherence of SR triad frequencies, quantifying non-linear coupling. **(F)** Theta-gamma phase-amplitude coupling (θ → γ PAC) dynamics across the event.

SIE events exhibited robust network synchronization, as shown in Figure 1. At the fundamental frequency SR1, 81.5% of events showed phase-locking values exceeding 0.6, and 60.5% showed magnitude-squared coherence above 0.5. Both MSC (0.552) and PLV (0.689) showed gradients decreasing from SR1 to SR6, with SR1 exhibiting highest synchronization—confirming whole-brain coordination during SIE events consistent with communication through coherence (Fries, 2015).

Power spectral elevation at SR harmonics was quantified using z-scores relative to session baselines. The fundamental frequency SR1 showed strongest elevation (*z* = 4.76 ± 2.95), with systematic decrease at higher harmonics, confirming multi-band spectral enhancement during SIE events.

#### 2.2.4 Temporal dynamics: the coherence-first signature

Individual SIE events exhibited a stereotyped six-phase temporal evolution:

1. Baseline: Low-to-moderate global synchronization (*R*≈0.4–0.6).
2. Coherence Rise: Rapid increase in phase alignment preceding amplitude changes, with *R* typically rising to >0.7 over 1–3 s.
3. Plateau: Brief stabilization (0.5–2 s) at elevated coherence (*R*>0.8) prior to amplitude ignition.
4. Ignition: Coincident peak in both coherence and power, with simultaneous z-score elevation across multiple harmonics.
5. Propagation: Sustained high-coherence state (5–15 s) with gradual spread of synchronization.
6. Decay: Gradual return to baseline over 3–8 s.

**Figure 2:**
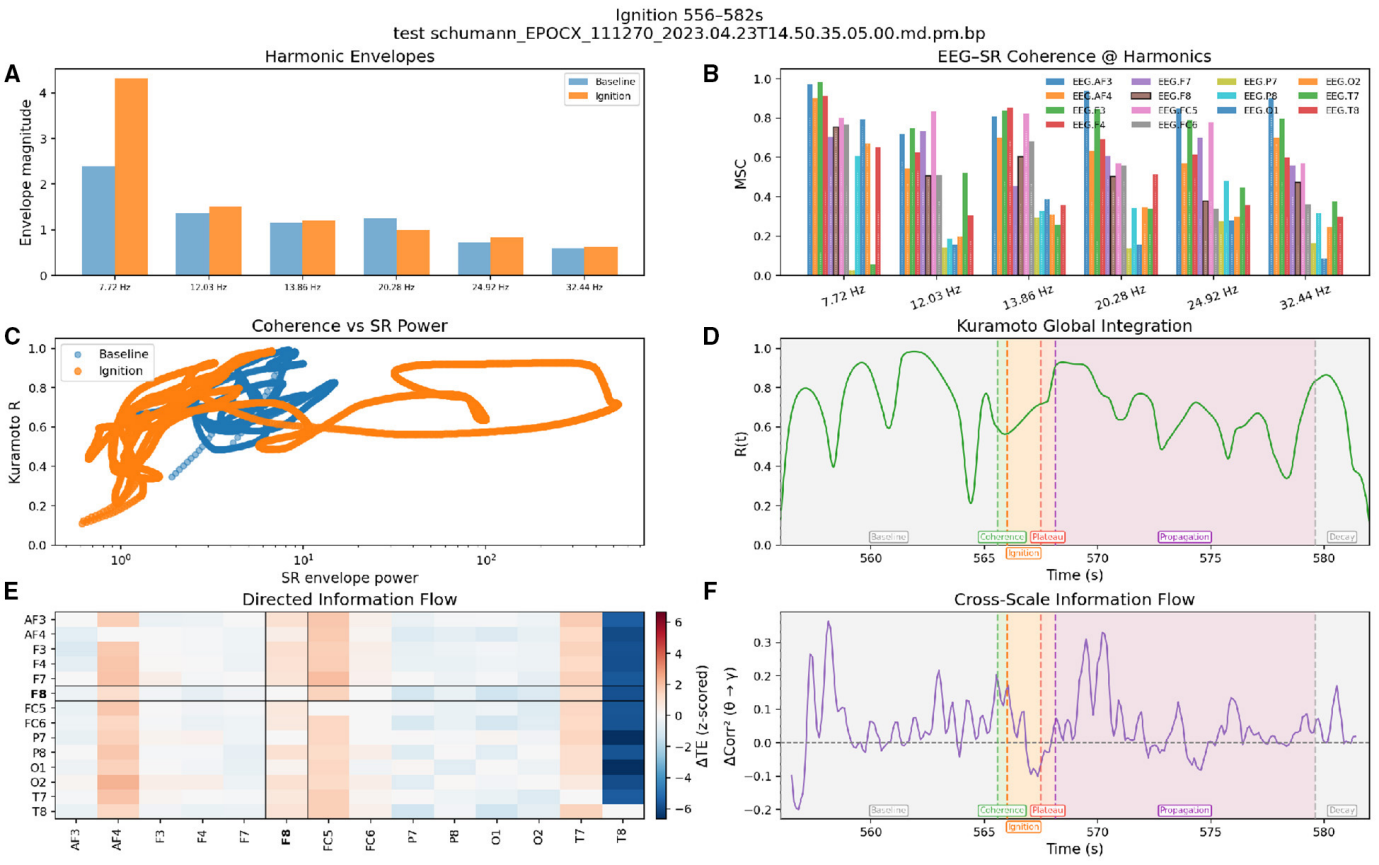
Exemplar SIE event network dynamics. **(A)** Harmonic envelope comparison between baseline and ignition states. **(B)** EEG-SR coherence by electrode and harmonic. **(C)** State-space trajectory showing coherence vs. power. **(D)** Kuramoto order parameter time series. **(E)** Transfer entropy matrix. **(F)** Cross-scale theta-gamma correlation dynamics.

The coherence-first signature—phase alignment preceding amplitude elevation by 2–3 s—is consistent with network-level coordination rather than local oscillatory bursts (Varela et al., 2001) (Figure 2).

#### 2.2.5 Cross-device and cross-context validation

One-way ANOVA tested whether harmonic frequencies differed across devices and contexts:

Device Independence: SR1 and SR3 showed no significant device differences (*p*>0.25), supporting biological rather than device-specific origins. SR5 exhibited a modest device effect (*F* = 5.74, *p* = 0.003), with Muse devices detecting slightly higher frequencies (32.88 Hz vs. 32.53–32.54 Hz for Emotiv devices)—likely reflecting differential hardware filtering at higher frequencies.

Context Independence: One-way ANOVA across five cognitive contexts (meditation, flow, non-flow, gaming, visual) revealed no significant frequency differences for any primary harmonic (all *p*>0.15). The φ^*n*^ architecture is preserved across contexts spanning eyes-closed meditation, cognitively demanding flow states, gaming engagement, and passive visual perception. This cross-context consistency across 91 participants argues against subject-specific effects; the same φ^*n*^ architecture emerges independently regardless of cognitive state or recording device.

#### 2.2.6 Individual subject replication

A potential concern is whether φ^*n*^ relationships emerge only in pooled data or reflect consistent patterns within individuals. Per-subject analysis of harmonic ratio precision addresses this directly. For each of the 90 subjects with valid harmonic triplets, the mean percentage deviation from theoretical φ^*n*^ values was computed (SR3/SR1 vs. φ^2^, SR5/SR1 vs. φ^3^, SR5/SR3 vs. φ). The top 10 subjects by event count are reported in Table 4.

**Table 4:** Per-subject φ^*n*^ ratio precision (top 10 by event count).

Subject	Dataset	Events	SR3/SR1	Err%	SR5/SR1	Err%	Mean Err%
muse_m1	muse	150	2.619	0.0	4.302	1.5	1.1
insight_m1	insight	48	2.583	1.3	4.213	0.6	0.9
files_m1	files	34	2.603	0.6	4.234	0.1	0.4
mpeng_507	mpeng	22	2.660	1.6	4.322	2.0	1.4
physf_s12	physf	22	2.634	0.6	4.275	0.9	0.7
physf_s14	physf	21	2.606	0.5	4.244	0.2	0.5
physf_s24	physf	20	2.586	1.2	4.201	0.8	0.9
physf_s22	physf	20	2.594	0.9	4.246	0.2	0.8
mpeng_540	mpeng	18	2.579	1.5	4.204	0.8	1.0
physf_s10	physf	18	2.591	1.0	4.218	0.4	0.7

Theoretical values: φ^2^ = 2.618 (SR3/SR1), φ^3^ = 4.236 (SR5/SR1). Error percentages show absolute deviation from theoretical.

Results demonstrate that φ^*n*^ precision is preserved within individuals, not merely in pooled statistics:

- Median per-subject error: 1.68% (mean: 2.14%).
- 62.2% of subjects showed < 2% mean ratio error.
- 94.4% of subjects showed < 5% mean ratio error.
- The three original discovery participants (files_m1, insight_m1, muse_m1) all exhibited < 1.1% error.

**Figure 3:**
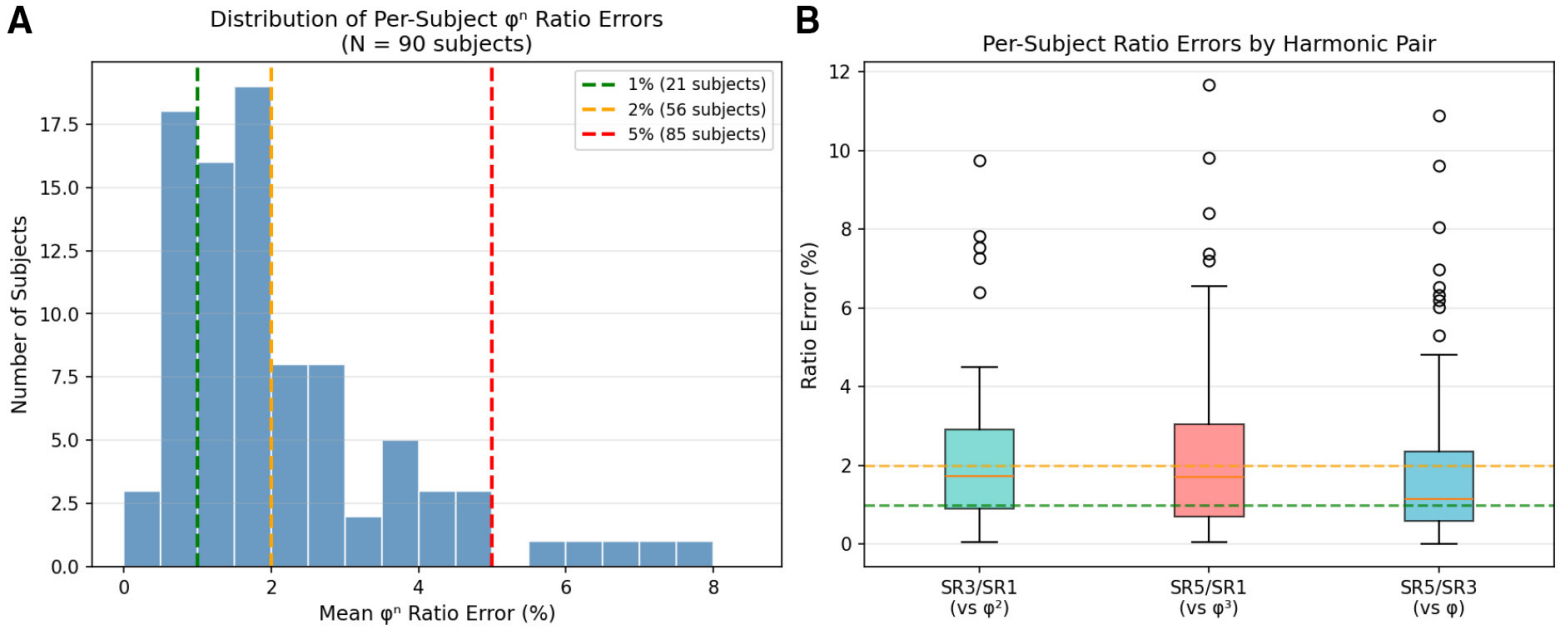
Per-subject φ^*n*^ ratio precision. **(A)** Distribution of mean ratio errors across 90 subjects, with vertical lines indicating 1%, 2%, and 5% error thresholds. The majority of subjects (62.2%) show < 2% deviation from theoretical φ^*n*^ values. **(B)** Box plots comparing per-subject error distributions for each harmonic ratio pair, demonstrating consistent precision across all three φ-predicted ratios.

Per-ratio error statistics across all 90 subjects: SR3/SR1 error = 2.16%±1.81%, SR5/SR1 error = 2.33%±2.23%, SR5/SR3 error = 1.94%±2.15%. The φ^*n*^ architecture is thus a robust individual-level phenomenon, not an artifact of inter-subject averaging (Figure 3).

#### 2.2.7 Session-level consistency and individual differences

To address whether φ^*n*^ ratio precision is maintained within individual recording sessions (not just in aggregate), per-session statistics were computed across all 513 sessions with valid harmonic triplets (Table 5).

**Table 5:** Per-session φ^*n*^ ratio precision summary.

Metric	SR3/SR1 (vs. φ^2^)	SR5/SR1 (vs. φ^3^)	SR5/SR3 (vs. φ)
Sessions analyzed	513	513	513
Mean error (%)	3.87	4.53	3.75
Median error (%)	3.20	3.71	3.13
Sessions < 2% error	33.5%	30.8%	34.1%
Within-session SD	0.118	0.206	0.066

Within-session SD computed for sessions with ≥2 events (*N* = 105).

Two-thirds of sessions (66.7%) showed mean ratio errors < 5%, with approximately one-third achieving < 2% precision. This demonstrates that φ^*n*^ organization is maintained at the session level, not merely an artifact of cross-session aggregation.

Three-level variance decomposition partitioned total variance in harmonic ratios into between-subject, between-session (within-subject), and within-session (event-level) components (Table 6).

**Table 6:** Variance decomposition of φ^*n*^ ratio precision.

Level	Description	SR3/SR1	SR5/SR1	SR5/SR3
Between-subject	Subject differences in mean adherence	12.1%	11.4%	8.0%
Between-session	Session variation within subjects	39.2%	43.2%	44.7%
Within-session	Event variation within sessions	48.8%	45.3%	47.3%

The variance decomposition reveals that only 8–12% of total variance occurs between subjects, with the majority distributed between sessions (39–45%) and within sessions (45–49%). This pattern—low between-subject variance with high within-session variance—is consistent with the independence-convergence paradox (Section 2.3.3): individual harmonic frequencies vary independently across events, yet aggregate ratios maintain φ^*n*^ precision through population-level constraints.

**Figure 4:**
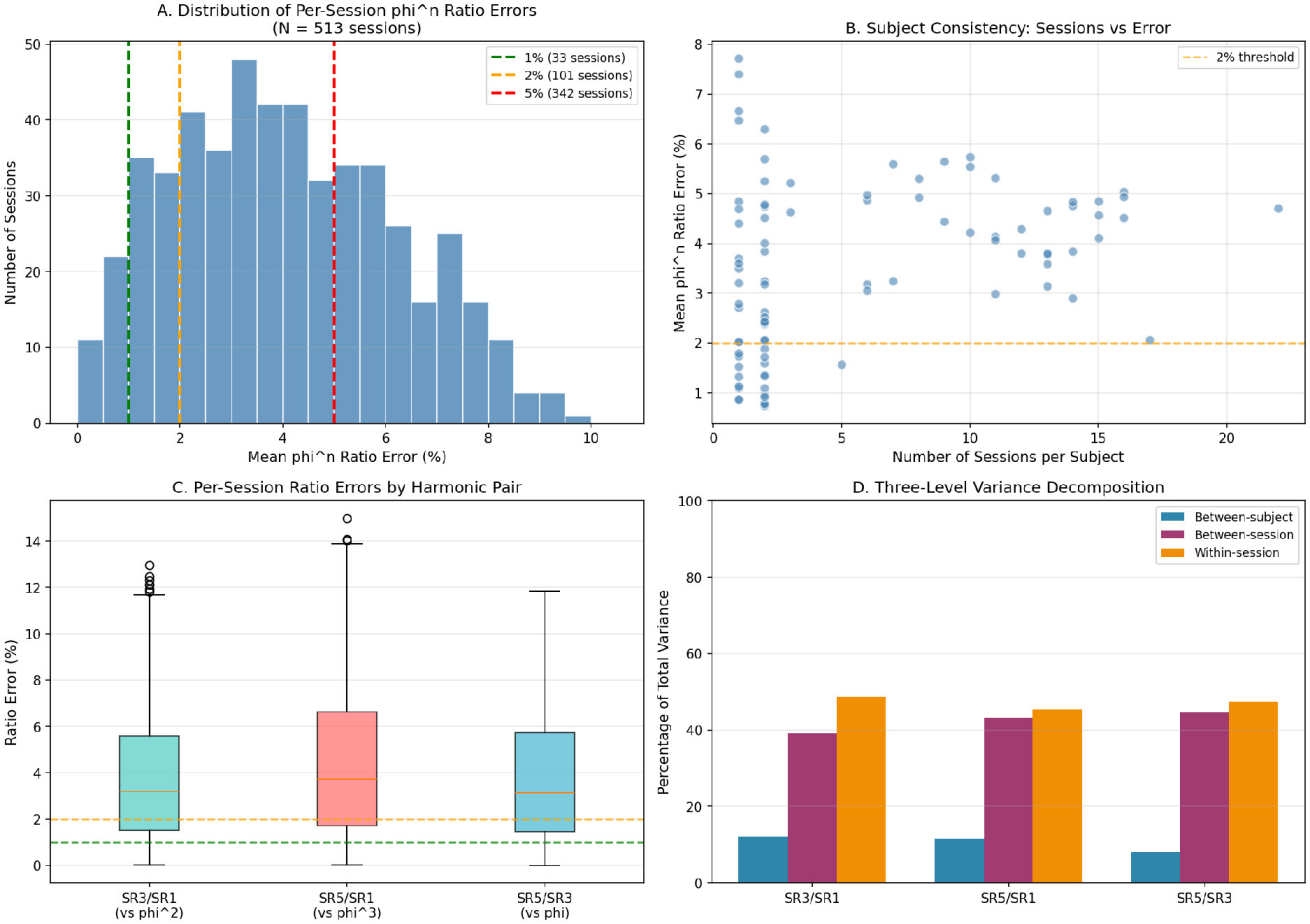
Per-session φ^*n*^ ratio analysis. **(A)** Distribution of per-session mean ratio errors across 513 sessions. **(B)** Relationship between number of sessions per subject and mean error, showing no systematic improvement with more data. **(C)** Box plots of session-level errors by ratio type. **(D)** Three-level variance decomposition showing that most variance is within-session (47–49%) and between-session (39–45%), with only 8–12% between subjects—consistent with population-level rather than individual-level constraints.

Intraclass correlation coefficients (ICC) confirmed this interpretation. Subject-level ICCs were uniformly low (SR3/SR1: 0.041 [95% CI: 0.011, 0.071]; SR5/SR1: 0.038 [0.005, 0.074]; SR5/SR3: −0.014 [−0.035, 0.011]), indicating that individual subjects do not show stable “high-adherence” or “low-adherence” profiles. Rather, φ^*n*^ architecture operates as a population-level constraint that emerges from the aggregate frequency distributions of each harmonic, not from consistent individual differences (Figure 4).

### 2.3 The emergent pattern: golden ratio frequency ratios

#### 2.3.1 The discovery

Upon examining the nine harmonic frequencies detected across events (Table 3), a striking pattern emerged: the measured frequencies align closely with a geometric series $f\left(n\right)=f_{0}\times \varphi^{n}$ where φ = 1.618034 (the golden ratio). Using an empirically-derived fundamental frequency *f*_0_ = 7.6 Hz, measured frequencies matched theoretical predictions across integer, half-integer, and quarter-integer values of *n*, with mean absolute error of only 1.5%.

#### 2.3.2 Golden ratio precision in harmonic ratios

The φ^*n*^ architecture predicts specific ratios between harmonics that are independent of *f*_0_ choice, providing the most stringent test of golden ratio organization (Table 7):

**Table 7:** Harmonic ratio precision: measured vs. φ^*n*^ predictions.

Ratio	Predicted	Measured	Error (%)	95% CI
SR3/SR1 = φ^2^	2.6180	2.6223 ± 0.134	+0.16	[2.614, 2.630]
SR5/SR1 = φ^3^	4.2361	4.2766 ± 0.249	+0.96	[4.262, 4.291]
SR5/SR3 = φ	1.6180	1.6309 ± 0.078	+0.79	[1.627, 1.635]
SR6/SR4 = φ	1.6180	1.6094 ± 0.100	−0.53	[1.604, 1.615]

Mean absolute ratio error: 0.61%. All four ratios deviated less than 1% from predicted φ^*n*^ values. Bootstrap analysis (*N* = 10,000 iterations) confirmed that the 95% confidence interval for SR3/SR1 (2.614–2.630) includes the predicted φ^2^ value (2.618).

#### 2.3.3 The independence-convergence paradox

A critical question emerged: Do the three primary harmonics (SR1, SR3, SR5) vary together proportionally, or independently? If frequencies were proportionally coupled, strong positive correlations across events would be expected. Instead, pairwise Pearson correlations revealed complete independence (Table 8):

**Table 8:** Frequency correlations across events: the independence-convergence paradox.

Pair	Pearson *r*	*p*-value	Interpretation
SR1 vs SR3	+0.030	0.33	No correlation
SR1 vs SR5	−0.002	0.94	No correlation
SR3 vs SR5	−0.020	0.47	No correlation

All correlations were statistically non-significant (all |*r*| < 0.03, all *p*>0.3), demonstrating that harmonic frequencies vary completely independently across events. An event with high SR1 frequency is no more likely to have high SR3 or SR5 frequencies than expected by chance.

**Key Insight**

**The Paradox:** Individual harmonic frequencies vary completely independently across events, yet their ratios maintain < 1% precision. This pattern cannot arise from coupled oscillators (which would show frequency covariation) nor from independent rhythms (which would degrade ratio precision).

**The Resolution:** φ^*n*^ relationships are encoded in the population-level marginal distributions of each harmonic rather than through event-level coordination. Each oscillatory generator is independently constrained to a frequency distribution whose mean satisfies φ^*n*^ relationships relative to other harmonics.

#### 2.3.4 Null control validation

The peak-based null control (Null Control D) provided the most stringent test. Comparing SIE frequency triplets against 10,000 random triplets sampled from actual FOOOF peaks (*N* = 244,955 peaks):

- Observed mean φ-error: 4.37%±2.00%.
- Random peak triplet mean error: 9.05%±4.15%.
- Cohen's *d*: 1.44 (large effect).
- *p*-value: < 0.0001 (permutation test).

**Figure 5:**
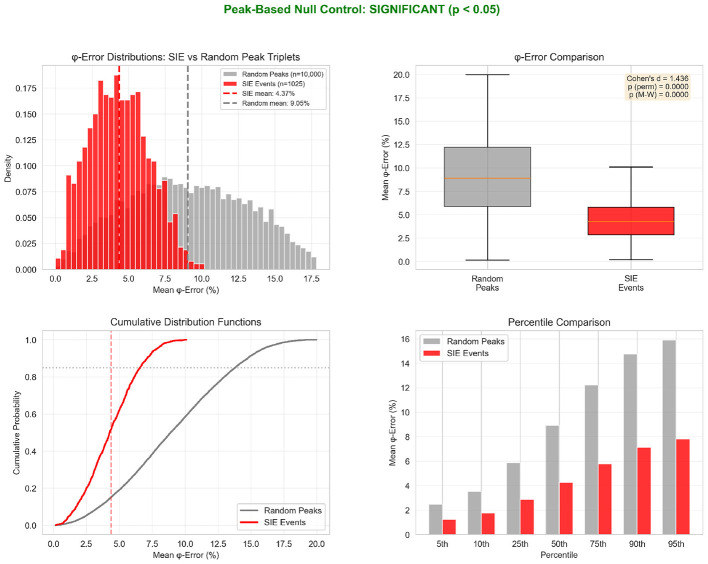
Peak-based null control results. The top-left panel shows a histogram comparing φ-error distributions for SIE events (red) versus random triplets sampled from actual FOOOF peaks (gray); SIE events cluster at lower φ-error values. The top-right panel shows a box plot comparison demonstrating significantly lower φ-error for SIE events. The bottom-left panel shows cumulative distribution functions illustrating separation between the two distributions. The bottom-right panel shows percentile comparison across the error distribution.

SIE events show significantly better φ-precision than random triplets sampled from actual EEG spectral peaks. The highly significant result (*p* < 0.0001) with large effect size (*d* = 1.44) indicates that SIE detection preferentially identifies frequency combinations exhibiting exceptional φ^*n*^ organization (Figure 5).

#### 2.3.5 Statistical power summary

Table 9 summarizes achieved statistical power for key analyses. Observed effect sizes substantially exceeded minimum detectable effects, confirming adequately powered conclusions.

**Table 9:** Statistical power for key analyses.

Analysis	*N*	Observed effect	Min detectable	Power
Ratio precision vs null	1,121	*d* = 1.44	*d* = 0.09	>0.999
Position enrichment	244,955	18–39%	< 1%	>0.999
Session attractor effect	968	*d* = 1.19	*d* = 0.09	>0.999
Frequency independence	1,121	|*r*| < 0.03	*r* = 0.08	0.20^*^
Device independence (SR5)	1,366	*F* = 5.74	*f* = 0.16	0.33

All analyses assumed α = 0.05 (two-tailed) and target power of 0.80 for sensitivity calculations.

^*^For frequency independence, low power at observed |*r*| < 0.03 confirms genuine independence rather than Type II error; there was 80% power to detect *r*≥0.08.

#### 2.3.6 The emergent question

The discovery of precise φ^*n*^ ratio organization in transient high-coherence events raised a fundamental question that motivated the second phase of this research:

*Is* φ^*n*^ *organization specific to transient SIE events, or does it reflect a deeper architectural principle governing* ***all*** *EEG frequency organization?*

If the latter, the φ^*n*^ boundary-attractor structure would be expected to be visible in the aggregate distribution of oscillatory peaks across the full spectrum, not only during SIE events. The theoretical framework and validation study presented in the following sections test this prediction.

The discovery of φ-ratio precision in SIE harmonics motivated the confirmatory analysis that follows. Critically, Study 2 employs unconstrained FOOOF spectral parameterization across the full frequency range—unlike Study 1's targeted search within Schumann Resonance frequency windows—providing an independent, hypothesis-testing evaluation of the φ^*n*^ framework.

## 3 Theoretical framework: the φ^*n*^ hypothesis

The discovery of precise golden ratio relationships in SIE harmonic frequencies (Section 2.3) raised a fundamental question: Does this organization reflect a deeper architectural principle governing all EEG frequency organization? The pattern was formalized into a testable theoretical framework.

**Critical Methodological Point**

**Attribution:** The theoretical framework for φ-ratio organization of neural oscillations was established by Pletzer et al. (2010), who proved mathematically that the golden ratio uniquely prevents spurious synchronization while enabling controlled coupling. The present study provides large-scale empirical validation of this framework and extends it by (1) identifying the absolute fundamental frequency *f*_0_ = 7.6 Hz, (2) characterizing the boundary-attractor structure in continuous EEG, and (3) discovering transient high-coherence states (SIEs) that represent amplified expression of the φ^*n*^ substrate.

### 3.1 Golden ratio mathematics and neural relevance

#### 3.1.1 Definition and properties

The golden ratio is defined as the positive solution to *x*^2^ = *x*+1 (Equation 6):


$$\begin{array}{l} \varphi =\frac{1+\sqrt{5}}{2}=1.6180339887... \end{array}$$ (6)

The golden ratio possesses two unique mathematical properties relevant to coupled oscillator systems:

Property 1: Maximal Anti-Commensurability. The golden ratio has the continued fraction representation φ = [1;1, 1, 1, ...], which converges more slowly than any other irrational. This means the rational approximations to φ (the Fibonacci ratios 1/1, 2/1, 3/2, 5/3, 8/5...) are the *worst possible* among all irrationals of comparable magnitude.

*Implication:* Coupled oscillators at frequency ratio φ maximally resist mode-locking. The phase relationship never settles into a stable pattern because φ cannot be well-approximated by any simple ratio. This enables segregation: oscillators at different φ^*n*^ positions maintain independence rather than collapsing into synchrony.

#### 3.1.2 Mathematical foundation: synchronization avoidance

Pletzer et al. (2010) provided the first rigorous mathematical proof that the golden ratio uniquely prevents spurious synchronization between neural oscillations. Their key results establish:

1. Mathematical impossibility: Two oscillations at frequency ratio φ can never synchronize in a mathematical sense—their excitatory phases meet at most once regardless of initial phase relationship.
2. Minimal physiological coupling: When phases do come close enough for physiological interaction (within threshold δ), these coincidences are *least frequent* and *most irregular* for φ among all possible frequency ratios.
3. Unique optimality: The golden mean is the “most irrational number”—its continued fraction expansion [1;1, 1, 1, ...] converges more slowly than any other irrational, making it maximally resistant to rational approximation and thus to mode-locking.
4. Noise robustness: These properties are preserved under stochastic perturbation, ensuring φ-organization remains stable in noisy neural environments.

This theoretical framework provides the mathematical foundation for why the brain would organize frequencies according to φ^*n*^: it is the *unique* solution maximizing desynchronization while permitting controlled, irregular coupling.

#### 3.1.3 Cross-frequency coupling: independent derivation of φ

While Pletzer et al. addressed synchronization *avoidance*, Kramer (2022) independently derived φ as the unique solution for cross-frequency *coupling*—addressing the complementary problem of how separate rhythms communicate when integration is required.

Using a damped harmonic oscillator model, Kramer showed that resonance between a driver oscillation (frequency *f*_*D*_) and target oscillation (*f*_*T*_) requires sinusoidal gain modulation at a specific frequency *f*_*S*_ satisfying Equation 7:


$$\begin{array}{l} f_{S}=f_{T}-f_{D},\;f_{S}=f_{D}-f_{T},\;\text{or }f_{S}=f_{D}+f_{T} \end{array}$$ (7)

The critical question is: for frequencies organized as $f_{k}=f_{0}\times c^{k}$, what value of *c* ensures the gain frequency *f*_*S*_ is *itself* a member of the original frequency set? Consider neighboring rhythms *f*_*k*_, *f*_*k*+1_, *f*_*k*+2_ where the gain frequency must couple two adjacent rhythms to the next. Using the additive condition (*f*_*S*_ = *f*_*D*_+*f*_*T*_) yields Equation 8:


$$\begin{array}{l} f_{k+2}=f_{k+1}+f_{k}\;\Rightarrow \;f_{0}c^{k+2}=f_{0}c^{k+1}+f_{0}c^{k}\\ \;\Rightarrow \;c^{2}-c-1=0 \end{array}$$ (8)

The unique positive solution is *c* = φ. This derivation establishes that φ is not merely “good” for neural organization—it is the *only* scaling factor enabling cross-frequency coupling where all participating frequencies (driver, target, and gain) belong to the same φ^*n*^ set. Alternative factors like Euler's number (*e*) require gain frequencies outside the original set; integer ratios (powers of 2) produce non-selective “coupling superhighways” (Hoppensteadt and Izhikevich, 1997).

*Convergence of independent derivations*. Pletzer et al. derived φ from synchronization avoidance; Kramer derived φ from cross-frequency coupling requirements. That both arrive at the same unique solution strengthens theoretical confidence: φ emerges from fundamentally different mathematical constraints as the singular solution for neural frequency organization.

*Resonance order hierarchy*. Building on Hoppensteadt and Izhikevich's theory of weakly coupled oscillators (Hoppensteadt and Izhikevich, 1997), Kramer introduced resonance order to quantify coupling strength. For frequencies satisfying $\underset{i}{\sum }k_{i}f_{i}=0$ (where *k*_*i*_ are integers), resonance order equals $\underset{i}{\sum }|k_{i}|$. Lower orders indicate stronger coupling. For golden triplets (*f*_*k*−1_+*f*_*k*_−*f*_*k*+1_ = 0), resonance order is 3—the minimum possible for cross-frequency interactions. This creates a hierarchical coupling structure where consecutive φ^*n*^ harmonics couple most strongly (order 3), with coupling strength decreasing for more distant harmonics. The observed SIE pattern (strong SR1-SR3-SR5 coupling) is consistent with this nearest-neighbor preference.

Property 2: Fibonacci Additivity. The golden ratio uniquely satisfies the recurrence in Equation 9:


$$\begin{array}{l} \varphi^{n}=\varphi^{n-1}+\varphi^{n-2}\;\text{for all }n\in \mathbb{Z} \end{array}$$ (9)

No other positive real number satisfies this property.

*Implication:* At frequency $f\left(n\right)=f_{0}\times \varphi^{n}$, the three frequencies *f*(*n*), *f*(*n*−1), and *f*(*n*−2) satisfy an exact sum relationship, enabling three-wave resonant energy transfer. This creates “gateways” for cross-frequency communication at specific positions, enabling integration.

**Key Insight**

Why φ May Be Unique for Neural Organization: No other scaling factor provides both properties. Integer ratios (2:1, 3:2) enable coupling but produce mode-locking; other irrationals ($\sqrt{2}$ *e*) resist mode-locking but lack Fibonacci coupling. Pletzer et al. (2010) proved mathematically that φ is the *unique* solution maximizing desynchronization while enabling controlled coupling—exactly the “segregation-integration balance” required for complex neural computation.

#### 3.1.4 Theoretical precedent: resting state organization

Pletzer et al. (2010) proposed that φ-ratio organization characterizes the *resting brain*, providing:

- Maximal desynchronization between frequency bands.
- Random, unpatterned, and rare phase coincidences.
- The possibility for spontaneous, irregular coupling and uncoupling.

They further suggested that active information processing involves transition to *harmonic (integer) ratios* (e.g., theta/upper alpha = 6:12 Hz = 2:1), which enable frequent, regular cross-frequency coupling. This theoretical distinction between φ-organized resting states and harmonically-organized active states provides important context for interpreting neural frequency architecture.

*Note:* While Pletzer et al.'s resting/active framework and the substrate-ignition model (Section 5.3) address related phenomena, they represent distinct conceptual approaches. Pletzer et al. focus on the mathematical properties of frequency ratios, while the present model addresses the temporal dynamics of transient high-coherence events. Both frameworks are consistent with φ^*n*^ as the fundamental organizing principle.

### 3.2 The core equation: $f\left(n\right)=f_{0}\times \varphi^{n}$

Based on the SIE discovery, the hypothesis that EEG frequencies are organized according to Equation 10 was formalized:

**Key Equation**

$$\begin{array}{l} f\left(n\right)=f_{0}\times \varphi^{n} \end{array}$$ (10)

where:

- *f*_0_ = 7.60 Hz (measured Schumann Resonance fundamental from Tomsk Observatory)
- φ = 1.6180339887... (golden ratio)
- *n* ∈ ℝ (continuous position index)

**Figure 6:**
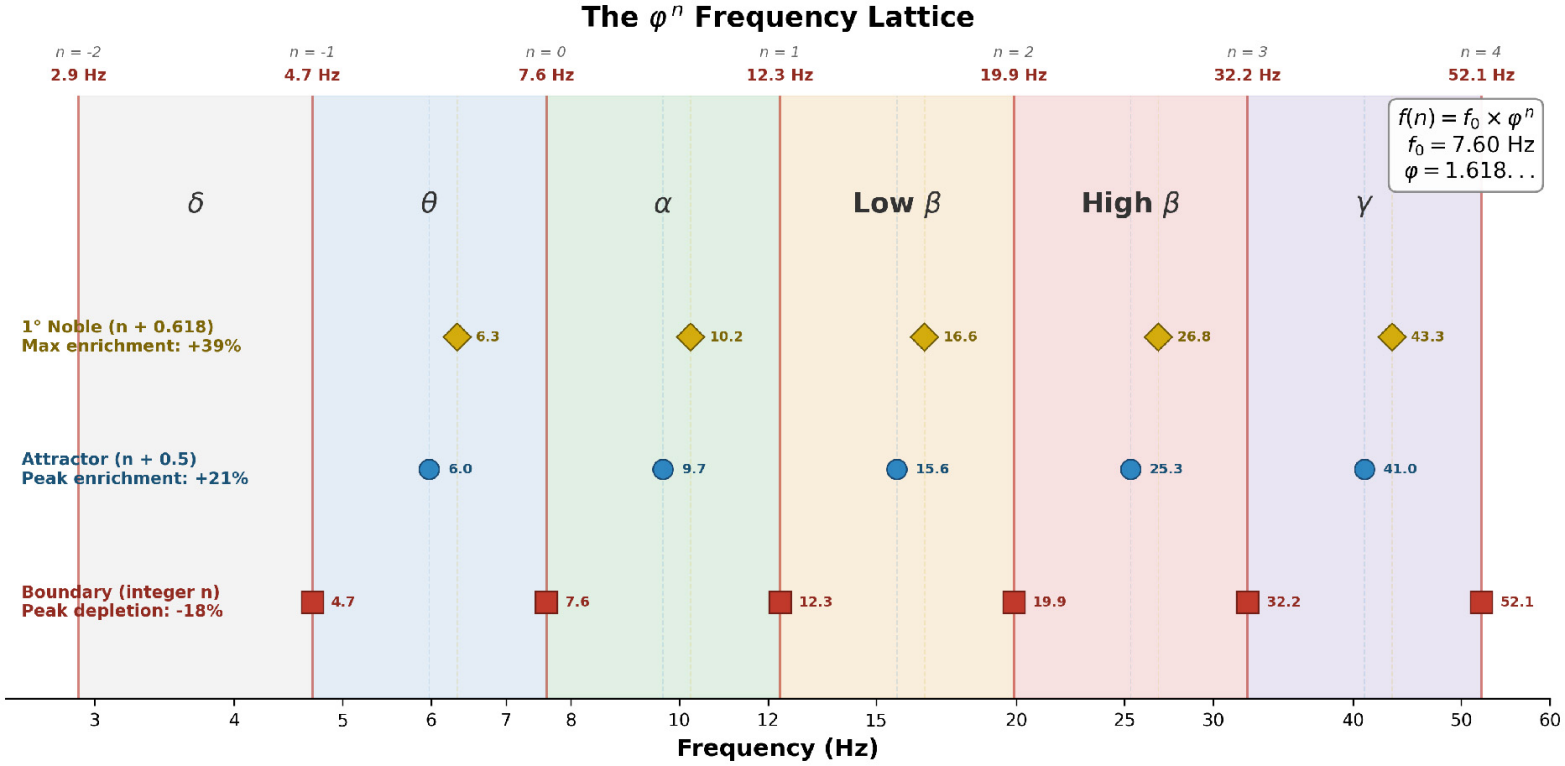
The φ^*n*^ frequency lattice. The equation $f\left(n\right)=f_{0}\times \varphi^{n}$ (where *f*_0_ = 7.60 Hz, φ = 1.618) generates a hierarchy of frequency positions that repeat within each φ-octave band. Solid red vertical lines mark band boundaries at integer *n* positions (*n* = −2 through *n* = 4), with frequencies labeled above. Within each band, three position types are shown: boundaries (red squares, bottom row; observed aggregate peak depletion −18%), attractors at half-integer *n* (blue circles, middle row; aggregate enrichment +21%), and 1° noble positions at *n*+0.618 (gold diamonds, top row; maximal aggregate enrichment +39%). These are cross-band aggregate values (see Table 21 note). Dashed vertical reference lines in matching colors highlight the regular spacing of attractor and noble positions across bands. Shaded regions indicate φ^*n*^-derived EEG band definitions from delta through gamma. All specific frequencies (Hz) are labeled to the right of each marker.

Figure 6 illustrates the resulting lattice and the position types it generates within each φ-octave band.

Convergent *f*_0_ Estimates. The fundamental frequency *f*_0_ = 7.60 Hz derives from two independent sources:

- Geophysical: Multi-year monitoring at Tomsk Space Observing System reports SR fundamental at 7.6 ± 0.2 Hz.
- Neural (SIE): Mean SR1 frequency across 1,121 SIE events was 7.63 ± 0.33 Hz.

The agreement within 0.03 Hz (0.4%) between independent neural and geophysical estimates provides mutual validation.

**Key Conceptual Claim**. The hypothesis concerns φ^*n*^ *ratio architecture*, not absolute frequency locking. Both Schumann Resonance (±0.3 Hz diurnal variation) and neural oscillations (individual differences, state fluctuations) are naturally variable. The claim is that ratio relationships (boundary depletion, attractor enrichment) persist across this natural variability—ratios, not frequencies, are the deep invariant.

**Theory-Experiment Convergence**. The φ^*n*^ architecture discovered empirically in SIE harmonic ratios (Section 2.3) converges with Kramer's (Kramer, 2022) independent mathematical derivation. Kramer arrived at φ as the unique solution to the cross-frequency coupling problem from first principles; φ^*n*^ organization was discovered empirically in neural data. This convergence from theory and experiment strengthens confidence in the framework.

### 3.3 Predictions for continuous spectral organization

#### 3.3.1 Position classification

The framework classifies positions by their *n* value:

The term “noble” derives from number theory, where noble numbers are those whose continued fraction expansion eventually becomes an infinite sequence of 1s—the same defining property of φ itself (Hardy and Wright, 1979). Just as φ is maximally resistant to rational approximation, the *n*+0.618 positions within each φ^*n*^ interval are maximally distant from both the bounding integer positions and the half-integer attractors, inheriting the parent φ's anti-mode-locking properties at a finer scale.

Why half-integer positions are stable: At a boundary (integer *n*), the Fibonacci property *f*(*n*) = *f*(*n*−1)+*f*(*n*−2) enables three-wave energy transfer, destabilizing sustained oscillations. Half-integer positions are maximally distant from these coupling points—oscillations “hide” from cross-frequency energy redistribution.

#### 3.3.2 The extended noble hierarchy

The complete noble hierarchy extends beyond the primary 1° and 2° nobles to include higher-order nobles and their *inverse* counterparts. This extension reveals a symmetric structure centered on the attractor (0.5) with profound implications for neural frequency organization.

Mathematical Identity of Inverse Nobles. While regular nobles are single powers of φ^−1^ (Table 10), inverse nobles are *sums* of φ^−*n*^ terms (Equations 11, 12):


$$\begin{array}{ll} 3^{•}\text{Inverse}:0.764 & =\varphi^{-1}+\varphi^{-4}=1-\varphi^{-3} \end{array}$$ (11)

$$\begin{array}{ll} 4^{•}\text{Inverse}:0.854 & =\varphi^{-1}+\varphi^{-3}=1-\varphi^{-4} \end{array}$$ (12)

This sum structure is key: inverse nobles inherit stability properties from *multiple* noble positions simultaneously, enabling multi-scale resonance pathways (Section 3.3.6).

**Table 10:** Extended position classification: complete noble hierarchy.

Position type	*n* value	φ^−*n*^ representation	Predicted behavior	Symmetric to
**Boundary**	Integer (*k*)	φ^0^ = 1	**Depletion**	—
4° Noble	*k*+0.146	φ^−4^	Weak enrichment	4° Inverse
3° Noble	*k*+0.236	φ^−3^	Weak enrichment	3° Inverse
2° Noble*	*k*+0.382	φ^−2^	Modest enrichment	1° Noble
**Attractor**	*k*+0.5	—	**Enrichment**	—
1°Noble*	*k*+0.618	φ^−1^	**Strong enrichment**	2° Noble
3° Inverse†	*k*+0.764	φ^−1^+φ^−4^	Strong enrichment	3° Noble
4° Inverse†	*k*+0.854	φ^−1^+φ^−3^	Strong enrichment	4° Noble

“Noble” positions derive from number theory: a number is *noble* if its continued fraction expansion eventually becomes all 1s (like φ = [1;1, 1, 1, ...]). The positions *k*+0.618 and *k*+0.382 are maximally “φ-like” within each unit cell.

[†] “Inverse nobles” are symmetric to regular nobles about the attractor (0.5). They can be expressed as sums of φ^−*n*^ terms: 0.764 = 1−φ^−3^ = φ^−1^+φ^−4^ and 0.854 = 1−φ^−4^ = φ^−1^+φ^−3^. This sum structure enables multi-scale resonance pathways. Bold values indicate the primary position types (Boundary, Attractor, 1° Noble) on which the empirical claims of the paper rest.

Symmetry About the Attractor. Noble positions form symmetric pairs about the central attractor (0.5), each pair equidistant from this midpoint (Table 11):

**Table 11:** Symmetric noble pairs about the attractor.

Lower position	Upper position	Distance from 0.5	Stability class
2° Noble (0.382)	1° Noble (0.618)	0.118	Highest
3° Noble (0.236)	3° Inverse (0.764)	0.264	High
4° Noble (0.146)	4° Inverse (0.854)	0.354	Moderate
Boundary (0.0)	Boundary (1.0)	0.500	Lowest

This symmetry explains why both members of each pair show similar enrichment patterns: they belong to the same stability class, reflected about the attractor. The complete frequency-by-position lookup across all φ-octave bands from sub-delta (*n* = −6) through gamma (*n* = 4), with the full 16-position hierarchy including 5°–7° nobles and inverses, is provided in Supplementary Tables S1–S10.

#### 3.3.3 Continued fraction analysis

Noble numbers are defined by continued fractions that eventually become all 1s—the defining property of φ itself. The continued fraction representations reveal why both regular and inverse nobles inherit anti-mode-locking properties:

Regular nobles (single φ^−*n*^ terms; Equations 13–15):


$$\begin{array}{l} 0.618=\left[0;1,1,1,1,\ldots \right]\;\left(\text{thegoldenratioitself}\right) \end{array}$$ (13)

$$\begin{array}{l} 0.382=\left[0;2,1,1,1,\ldots \right]\;\left(\text{reaches“all}1{\text{s}}^{\prime \prime }\text{afteronestep}\right) \end{array}$$ (14)

$$\begin{array}{l} 0.236=\left[0;4,4,1,1,\ldots \right]\;\left(\text{takeslongertostabilize}\right) \end{array}$$ (15)

Inverse nobles (sums of φ^−*n*^ terms; Equations 16, 17):


$$\begin{array}{l} 0.764=\left[0;1,3,1,1,1,\ldots \right]\;\left(\text{reaches“all}1{\text{s}}^{\prime \prime }\text{quickly}\right) \end{array}$$ (16)

$$\begin{array}{l} 0.854=\left[0;1,5,1,1,1,\ldots \right]\;\left(\text{reaches“all}1{\text{s}}^{\prime \prime }\text{quickly}\right) \end{array}$$ (17)

The critical observation is that inverse nobles have continued fractions that *rapidly converge* to the golden pattern [..., 1, 1, 1, ...]. In dynamical systems terms, they inherit the anti-mode-locking properties of φ almost as strongly as φ itself. This rapid convergence explains why inverse nobles show strong enrichment comparable to primary nobles.

#### 3.3.4 Resonance stability and KAM theory

In KAM (Kolmogorov-Arnold-Moser) theory and coupled oscillator systems, stability depends on how *poorly* a frequency ratio is approximated by rationals. For ratio ω, the “resonance denominator” quantifies coupling strength as in Equation 18:


$$\begin{array}{l} \text{Couplingstrength}\propto \frac{1}{|p-q\omega {|}^{2}} \end{array}$$ (18)

where *p*/*q* is the nearest rational approximation.

Positions that require large denominators for rational approximation are maximally resistant to mode-locking (Table 12):

**Table 12:** Rational approximation and resonance stability.

Position	Value	Nearest simple rational	Denominator	Relative stability
Boundary	0, 1	0/1, 1/1	1	LOWEST
4° Noble	0.146	1/7	7	Medium
3° Noble	0.236	1/4	4	Medium
2° Noble	0.382	2/5	5	High
Attractor	0.500	1/2	2	Medium-High
1° Noble	0.618	3/5, 5/8, 8/13	Fibonacci	HIGHEST
3° Inverse	0.764	3/4, 13/17	4, 17	High
4° Inverse	0.854	5/6, 6/7	6, 7	Medium-High

The 1° noble (0.618) is hardest to approximate—it requires Fibonacci ratios (3/5, 5/8, 8/13, ...) which have the worst approximation properties among all irrationals. The inverse nobles require moderately large denominators, making them resistant to mode-locking while remaining accessible for controlled coupling.

#### 3.3.5 Cross-frequency coupling geometry

For an oscillator at position *u* within a φ^*n*^ band (where 0 ≤ *u* < 1), coupling risk to adjacent bands depends asymmetrically on position (Equations 19, 20):


$$\begin{array}{ll} \text{Lower}-\text{bandcouplingrisk} & \propto |u{|}^{-1} \end{array}$$ (19)

$$\begin{array}{ll} \text{Upper}-\text{bandcouplingrisk} & \propto |1-u{|}^{-1} \end{array}$$ (20)

These asymmetric coupling risks are summarized for each position type in Table 13.

**Table 13:** Asymmetric coupling risk by position.

Position	*u*	Lower risk	Upper risk	Dominant risk
4° Noble	0.146	HIGH	Low	Lower boundary
3° Noble	0.236	Medium	Low	Lower boundary
2° Noble	0.382	Balanced	Balanced	Neither
Attractor	0.500	Balanced	Balanced	Neither
1° Noble	0.618	Low	Medium	Upper boundary
3° Inverse	0.764	Low	LOW	Protected from upper
4° Inverse	0.854	Low	LOWEST	Protected from upper

Bold values (rendered in capitals) indicate extreme values or categorical a priori predictions.

This asymmetry explains a key functional prediction: when neural oscillators need to avoid coupling to higher-frequency bands while permitting downward coupling, they should preferentially occupy inverse noble positions. This has direct implications for theta-gamma phase-amplitude coupling, where theta oscillations must couple *upward* to gamma (for PAC) while avoiding mode-locking to alpha (see Section 6.2.3).

#### 3.3.6 Fibonacci sum property and multi-scale pathways

The inverse nobles possess a unique property arising from the Fibonacci recurrence given in Equation 21. Since:


$$\begin{array}{l} \varphi^{-n}=\varphi^{-\left(n+1\right)}+\varphi^{-\left(n+2\right)} \end{array}$$ (21)

the position 0.764 = φ^−1^+φ^−4^ can be decomposed via Fibonacci cascades (Equations 22–25):


$$\begin{array}{l} 0.764 \end{array}$$

$$\begin{array}{ll} = & \varphi^{-1}+\varphi^{-4} \end{array}$$ (22)

$$\begin{array}{ll} = & \left(\varphi^{-2}+\varphi^{-3}\right)+\varphi^{-4}\;\left[\text{Fibonacciexpansionof}{\text{φ}}^{-1}\right] \end{array}$$ (23)

$$\begin{array}{ll} = & \varphi^{-2}+\left(\varphi^{-4}+\varphi^{-5}\right)+\varphi^{-4}\left[\text{Fibonacciexpansionof}{\text{φ}}^{-3}\right] \end{array}$$ (24)

$$\begin{array}{ll} = & \varphi^{-2}+2\varphi^{-4}+\varphi^{-5} \end{array}$$ (25)

This decomposition creates multi-scale resonance pathways: an oscillator at the 3° inverse noble position has direct resonance pathways to:

- 1° noble (via the φ^−1^ component).
- 4° noble (via the φ^−4^ component).
- 2° noble (indirect, via Fibonacci cascade).

This enables controlled multi-band integration without the instability of boundary positions. The inverse nobles thus serve as “integration hubs”—positions that can participate in cross-frequency coupling across multiple scales while maintaining independence from immediate neighbors.

#### 3.3.7 A priori frequency landmarks

Specific frequency predictions generated from the framework before data analysis are summarized in Table 14.

**Table 14:** A priori frequency predictions (generated before data analysis).

*n*	φ^*n*^	*f*(*n*) Hz	Type	Predicted peak count
−1	0.618	4.70	Boundary	LOW (δ/θ border)
−0.5	0.786	5.97	Attractor	ELEVATED (θ center)
0	1.000	7.60	Boundary	LOW (θ/α border)
**+0.5**	1.272	**9.67**	**Attractor**	**HIGH (α** **peak)**
+1	1.618	12.30	Boundary	LOW (α/β border)
+1.5	2.058	15.64	Attractor	ELEVATED (low β)
+2	2.618	19.90	Boundary	LOW or ELEVATED*
+2.5	3.330	25.31	Attractor	ELEVATED (high β)
**+3**	4.236	**32.19**	**Boundary**	**LOW (β/γ** **trough)**
**+3.5**	5.388	**40.95**	**Attractor**	**HIGH (γ** **peak)**
+4	6.854	52.09	Boundary	LOW (high γ border)

^*^The φ^2^ position may show resonance enhancement due to Fibonacci three-wave coupling. Bold values (rendered in capitals) indicate extreme values or categorical a priori predictions.

#### 3.3.8 Predicted band-specific heterogeneity

While the φ^*n*^ architecture is predicted across all frequency bands, frequency-dependent expression strength is expected:

- Gamma band (φ^3^–φ^4^; 32–52 Hz): Should show the *strongest* φ^*n*^ alignment. Gamma oscillations subserve temporal binding and feature integration, requiring precise phase relationships within ~25 ms windows. The GABA-A receptor decay constant (τ≈25 ms) creates a natural oscillatory bottleneck near 40 Hz. The observed strict φ^*n*^ compliance is consistent with requirements for gamma oscillator independence, though the mechanism linking GABA-A kinetics to φ^*n*^ positioning remains to be established.
- Alpha band (φ^0^–φ^1^; 7.6–12.3 Hz): Should show *moderate* alignment, potentially obscured by individual alpha frequency variability (±1 Hz across subjects). Alpha functions primarily as a gating mechanism, which may tolerate broader frequency flexibility.
- Theta/Delta bands (< φ^0^): Should show the *weakest* alignment. These slower oscillations support memory consolidation and homeostatic functions that integrate information over longer timescales, permitting greater frequency variability.

This gradient predicts that φ^*n*^ architecture manifests as a universal position hierarchy (1° Noble > Attractor > 2° Noble) with frequency-dependent magnitude, with gamma predicted to show strongest expression and lower-frequency bands progressively weaker, though the aggregate analysis in Section 4.2.4 may conflate distinct band-specific patterns (see Limitation 5, Section 6.8).

## 4 Study 2: testing the φ^*n*^ hypothesis in continuous EEG

The theoretical framework derived from SIE discovery (Section 3) generated specific predictions for continuous spectral organization. If φ^*n*^ architecture is fundamental rather than event-specific, aggregate peak distributions should show boundary depletion, attractor enrichment, and the predicted noble position hierarchy.

### 4.1 Methods: spectral parameterization and analysis

#### 4.1.1 Extended dataset

The analysis utilized the same recording sessions as Study 1, extended to include all available EEG data regardless of SIE detection. The complete dataset comprised 968 sessions from approximately 96 unique subjects (91 from Study 1 plus additional sessions from shared devices), totaling 34.2 h of recording across three device types and five cognitive contexts.

#### 4.1.2 Independent replication dataset: EEGEmotions-27

To test generalizability beyond meditation and cognitive task contexts, spectral analysis was extended to the EEGEmotions-27 dataset (Phuong et al., 2025): 612,990 FOOOF-detected peaks across 2,342 sessions recorded during emotion induction paradigms using Emotiv EPOC X devices (256 Hz sampling rate, 14 electrodes). This dataset provides an independent test of whether φ^*n*^ organization persists in affect-driven neural states.

#### 4.1.3 FOOOF spectral parameterization

FOOOF (Fitting Oscillations and One-Over-F) (Donoghue et al., 2020) was employed for spectral parameterization. FOOOF separates the power spectrum into aperiodic and periodic components as in Equation 26:


$$\begin{array}{l} P\left(f\right)=L\left(f\right)+\underset{i}{\sum }G_{i}\left(f\right) \end{array}$$ (26)

where *L*(*f*) = *b*−log(*f*^χ^) is the aperiodic component and *G*_*i*_(*f*) are Gaussian peaks with center frequency μ_*i*_, amplitude *a*_*i*_, and bandwidth σ_*i*_.

FOOOF parameters: frequency range 1–50 Hz, peak width limits 0.2–20.0 Hz, maximum 20 peaks, minimum peak height 0.0001 log units. Peaks were retained if power > 0.1 log units above aperiodic fit, bandwidth 1–8 Hz, and frequency within 1–48 Hz.

#### 4.1.4 Lattice coordinate analysis

To analyze peaks independent of band-specific effects, the lattice coordinate for each peak was computed via Equation 27:


$$\begin{array}{l} u=\left[\log_{\varphi }\left(f/f_{0}\right)\right]\;\mathrm{mod}\;1 \end{array}$$ (27)

This maps all frequencies to the unit interval [0, 1), where *u* = 0 corresponds to boundaries, *u* = 0.5 to attractors, and *u* = 0.618 to primary noble positions.

### 4.2 Results: position-type enrichment and band structure

#### 4.2.1 Peak detection results

**Figure 7:**
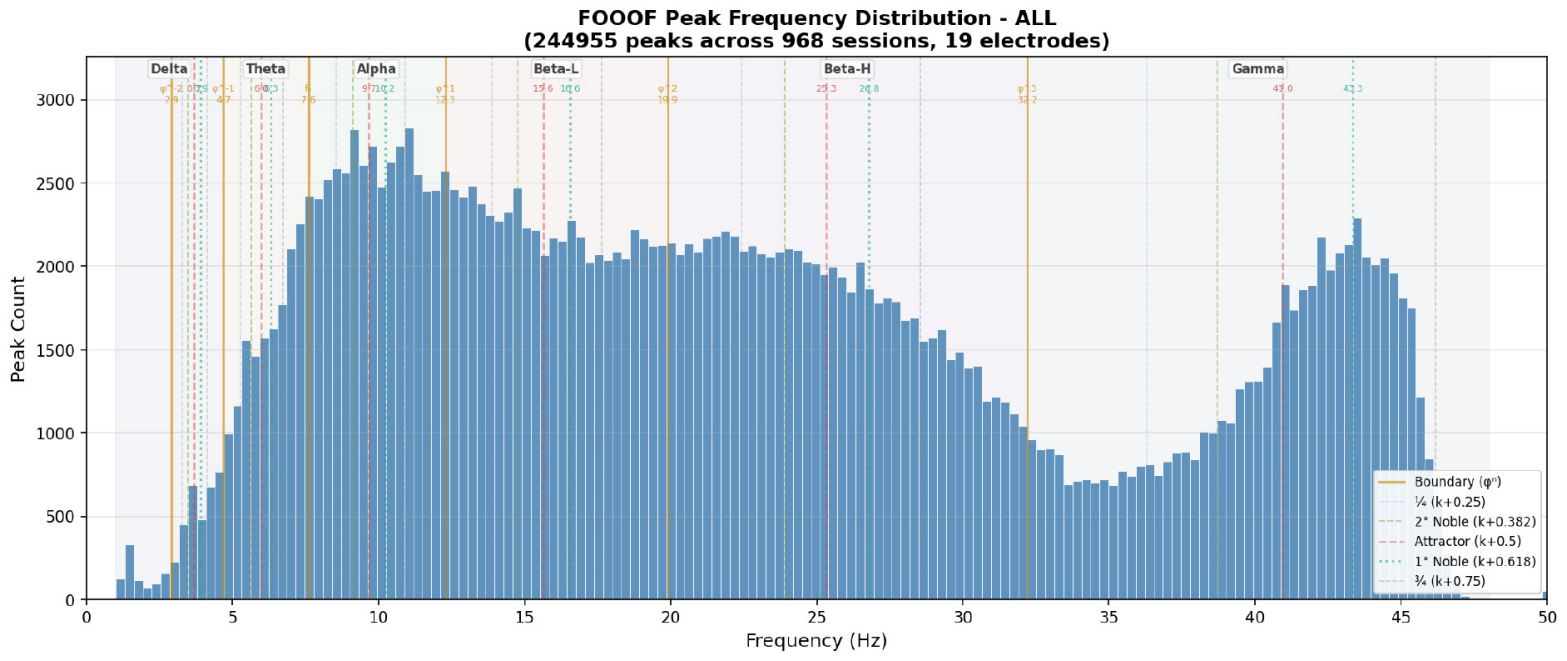
Distribution of 244,955 spectral peaks detected via FOOOF across 968 sessions in the primary dataset. Vertical lines indicate φ^*n*^ frequency predictions: boundaries at integer *n* (solid orange), attractors at half-integer *n* (dashed red), 1° nobles at *n*+0.618 (dotted teal), and 2° nobles at *n*+0.382 (dashed olive). Shaded regions demarcate traditional EEG bands.

**Figure 8:**
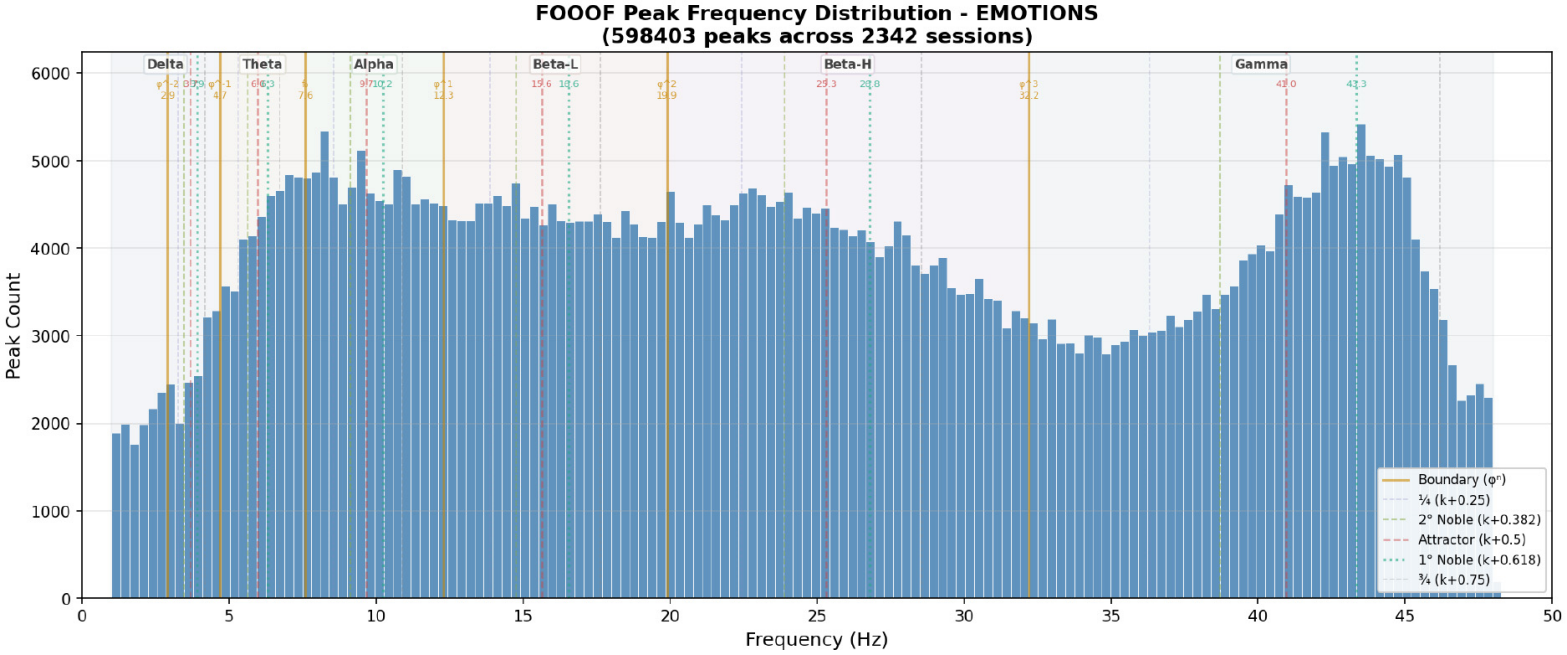
Distribution of 612,990 spectral peaks detected via FOOOF across 2,342 sessions in the EEGEmotions-27 dataset. The distribution shows similar bimodal structure to the primary dataset (Figure 7), with peaks in alpha (~10 Hz) and beta-gamma (~20–40 Hz) ranges. Vertical lines indicate φ^*n*^ frequency predictions as in Figure 7.

FOOOF parameterization yielded 244,955 oscillatory peaks across 968 sessions in the primary dataset, with a median peak frequency of 21.2 Hz and mean of 18.5 peaks per channel (Figure 7). The independent EEGEmotions-27 dataset yielded 612,990 peaks across 2,342 sessions with similar spectral structure (Figure 8). The complete peak inventory with frequency, power, bandwidth, channel, session, and dataset metadata is provided in Supplementary Data Sheet 2.

#### 4.2.2 Position-type enrichment

**Figure 9:**
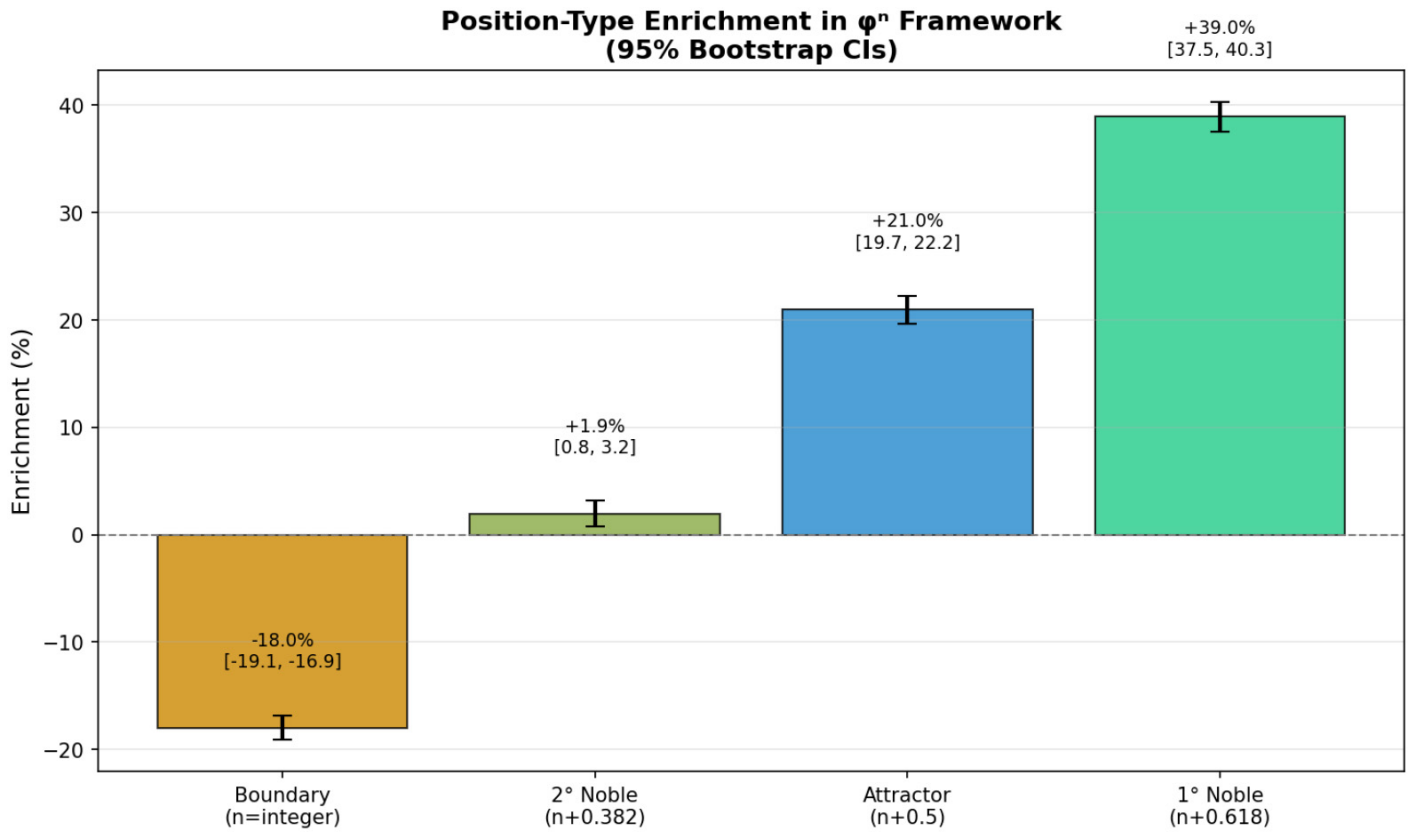
Position-type enrichment in the φ^*n*^ framework with 95% bootstrap confidence intervals (*n* = 243, 704 peaks after quality filtering). Bars show percent deviation from uniform expectation. Boundaries (integer *n*): −18.0% [CI: −19.1% to −17.0%]. 1° nobles (*n*+0.618): +39.0% [CI: +37.7% to +40.4%]. Attractors (*n*+0.5): +21.0% [CI: +19.7% to +22.4%]. The monotonic ordering (Boundary < 2° Noble < Attractor < 1° Noble) confirms theoretical predictions.

The aggregate peak distribution was analyzed for the extended 8-position hierarchy (see Section 3.3.2):

The core hierarchy (Boundary < 2° Noble < Attractor < 1° Noble) confirmed theoretical predictions (Kendall's τ = 1.0, *p* = 0.042; Figure 9). (Note: this ordering reflects the aggregate cross-band analysis; individual frequency bands may show distinct position preferences, as suggested by the band-specific heterogeneity in Table 15. The inverse nobles showed aggregate depletion (−2.7% and −17.1%), which reflects band-specific heterogeneity: as shown in Section 6.2.3, inverse nobles are *enriched* in theta but strongly *depleted* in gamma, yielding net depletion when aggregated across bands.

**Table 15:** Band-specific 1° noble enrichment.

Band	*n* range	Peaks	1°Noble enrichment
Delta	φ^−2^ to φ^−1^	3,140	−12.1%
Theta	φ^−1^ to φ^0^	15,315	+2.2%
Alpha	φ^0^ to φ^1^	39,189	+4.2%
Low Beta	φ^1^ to φ^2^	54,709	+3.9%
High Beta	φ^2^ to φ^3^	71,641	+8.8%
**Gamma**	φ^3^ to φ^4^	58,774	**+144.8%**

Aggregate cross-band values at the 1° Noble position. Within-band position structure may differ (see Limitation 5). Bold values indicate the strongest observed band-specific effect (Gamma at 1° Noble).

Cross-Dataset Replication: Independent analysis of the EEGEmotions-27 dataset confirmed the core φ^*n*^ hierarchy across diverse paradigms (Table 16).

**Table 16:** Position-type enrichment: two-dataset comparison.

Position type	Primary (N=244,955)	EEGEmotions-27 (*N* = 612,990)
Boundary	−18.0%	−20.2%
2° Noble	+1.9%	−1.0%
Attractor	+21.0%	+14.5%
1° Noble	+39.0%	+27.3%
Kendall's τ	1.0	1.0
Optimal *f*_0_	7.60 Hz	7.80 Hz
Session consistency	87.4%	83.7%

Critical finding: The core hierarchy (1° Noble > Attractor > 2° Noble > Boundary) replicated with Kendall's τ = 1.0 across both datasets spanning meditation and emotion induction paradigms. Effect magnitudes showed moderate attenuation from Primary (+39% 1° Noble) to EEGEmotions-27 (+27%), potentially reflecting paradigm-specific neural dynamics.

#### 4.2.3 Alignment with predicted landmarks

Key spectral landmarks aligned precisely with φ^*n*^ predictions:

- Alpha peak: Observed 9.8 Hz, predicted φ^0.5^ = 9.67 Hz (error: +0.13 Hz).
- Beta-gamma trough: Observed 32.4 Hz, predicted φ^3^ = 32.19 Hz (error: +0.21 Hz).
- Gamma recovery: Observed 41.2 Hz, predicted φ^3.5^ = 40.95 Hz (error: +0.25 Hz).

Mean absolute prediction error: 0.17 ± 0.09 Hz across all positions.

#### 4.2.4 Band-specific heterogeneity

**Figure 10:**
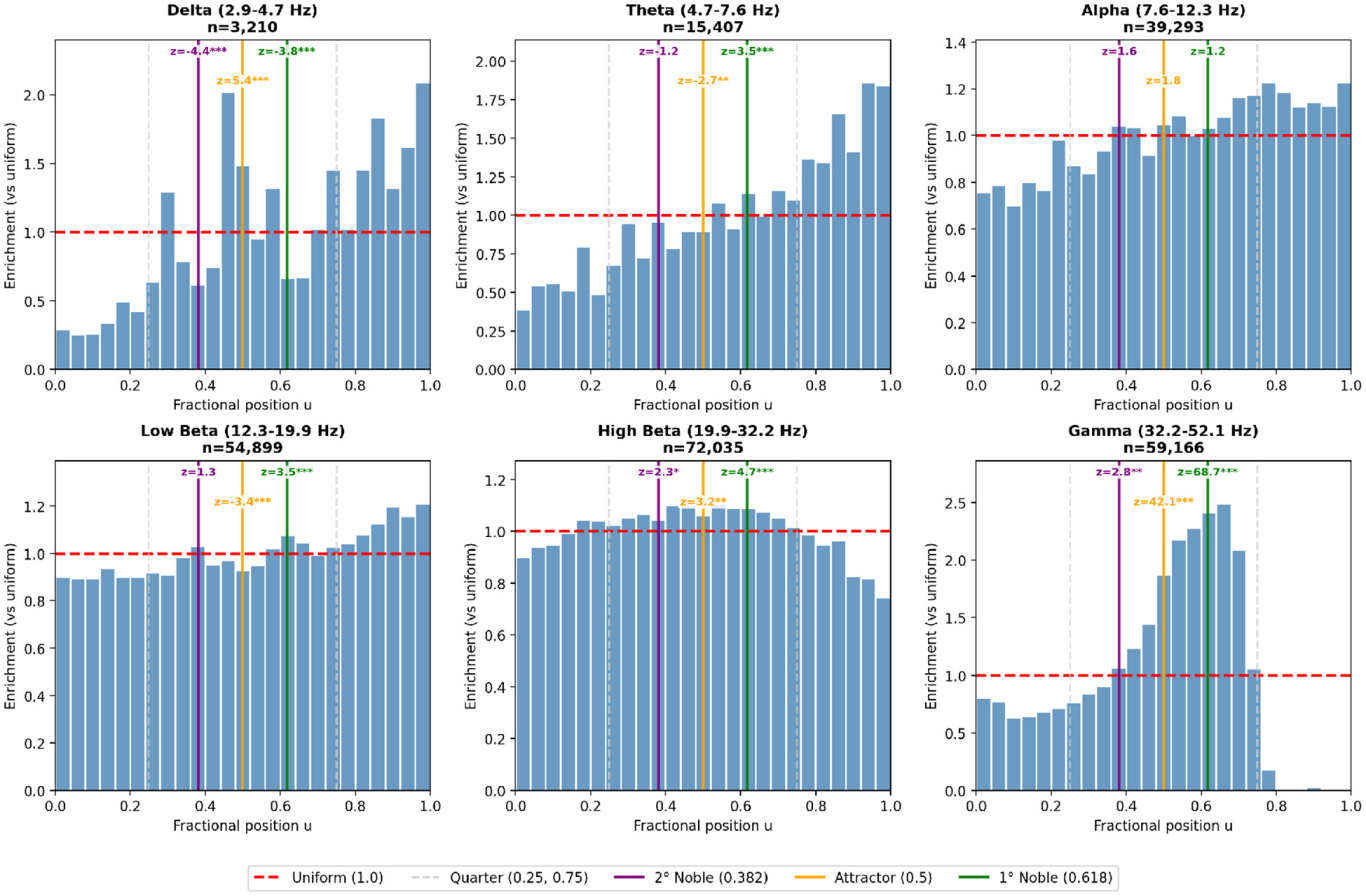
Band-stratified lattice coordinate analysis. Six panels show histograms of the fractional lattice position *u* = [log_φ_(*f*/*f*0)] mod 1 within each φ^*n*^-defined frequency band. Vertical lines mark theoretically significant positions. Gamma shows the strongest 1° noble enrichment (+145%), while alpha shows weak effects due to individual alpha frequency variability.

Stratified analysis by φ^*n*^-defined bands revealed frequency-dependent enrichment strength, confirming the predicted gradient:

Gamma dominance: The gamma band showed dramatically stronger aggregate φ^*n*^ adherence (+144.8% at 1° noble) than any other band in this cross-band analysis (Figure 10). This pattern is consistent with gamma's functional requirements: temporal binding and feature integration within ~25 ms windows demand precise phase relationships (Bartos et al., 2007), and φ^*n*^ organization may provide the anti-mode-locking properties needed to maintain independent gamma generators. Lower-frequency bands serve functions tolerating greater flexibility. As noted in Limitation 5, aggregate enrichment values are sensitive to spectral parameterization methodology and may not fully capture within-band position structure.

### 4.3 Validation: cross-device and cross-context consistency

#### 4.3.1 Alternative scaling factor comparison

To test whether the observed spectral organization is specific to φ or could arise from any exponential scaling, enrichment analysis was repeated using six candidate scaling factors: φ (1.618), 2 (octave), π (3.142), *e* (2.718), 1.5, and $\sqrt{2}$ (1.414). For each factor *c*, the lattice coordinate *u* = [log_*c*_(*f*/*f*_0_)] mod 1 was computed and position-type enrichment evaluated using the same boundary-attractor-noble framework.

**Figure 11:**
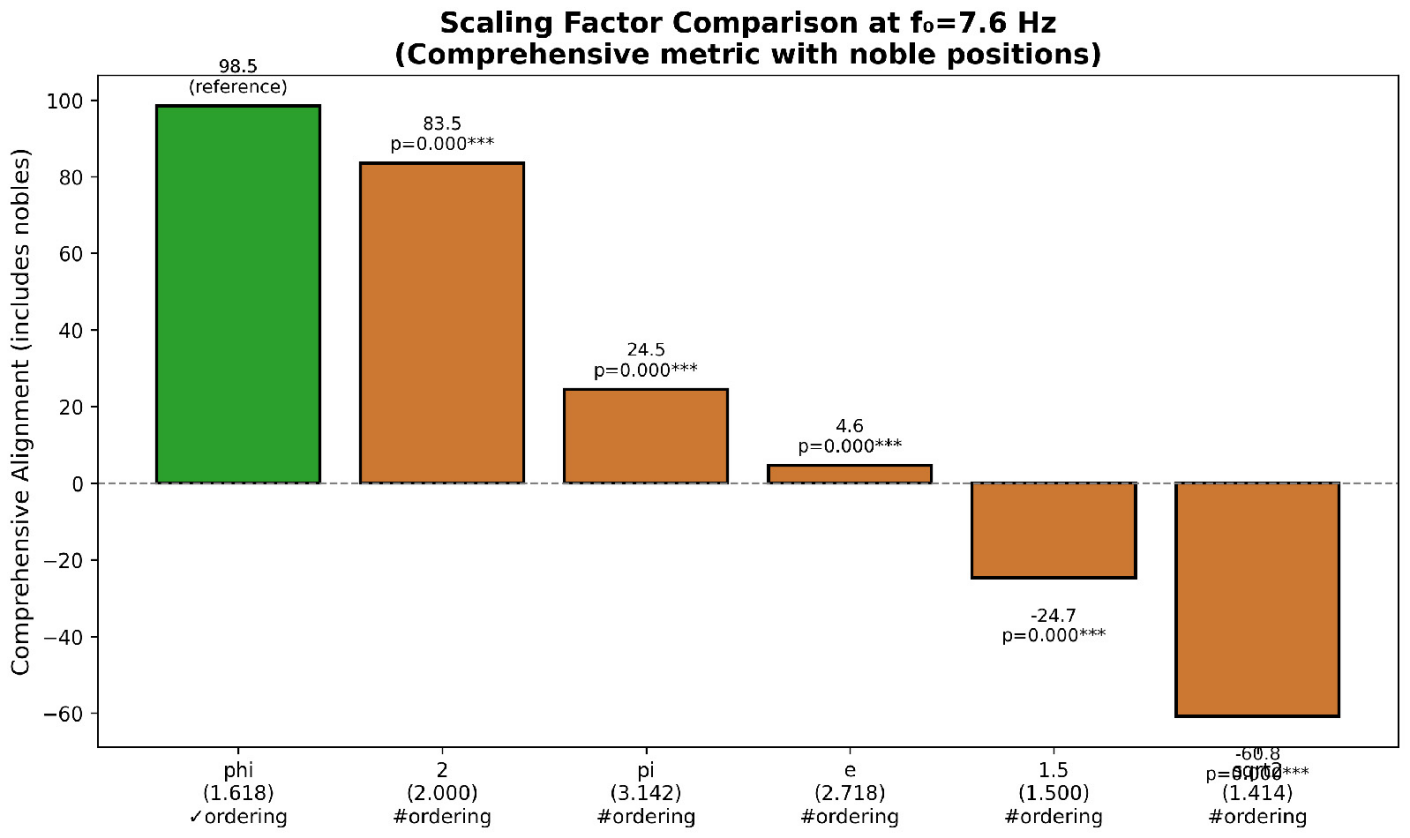
Alternative scaling factor comparison at *f*_0_ = 7.6 Hz. Comprehensive alignment metric (including noble position enrichment) for six candidate scaling factors. Only φ (green) achieves the theoretically predicted position-type ordering (✓ordering); all alternatives fail this test (#ordering). φ achieves the highest score (98.5), followed by base 2 (83.5), with rational bases (1.5, $\sqrt{2}$) showing negative alignment.

Only φ produced the theoretically predicted position-type ordering (1° Noble > Attractor > 2° Noble > Boundary; Kendall's τ = 1.0). Base 2 achieved the second-highest comprehensive alignment score (83.5 vs. 98.5 for φ), but failed the ordering test. All other scaling factors showed substantially lower alignment or negative scores, indicating anti-correlation with their respective lattice positions (Figure 11).

#### 4.3.2 Session-level consistency

**Figure 12:**
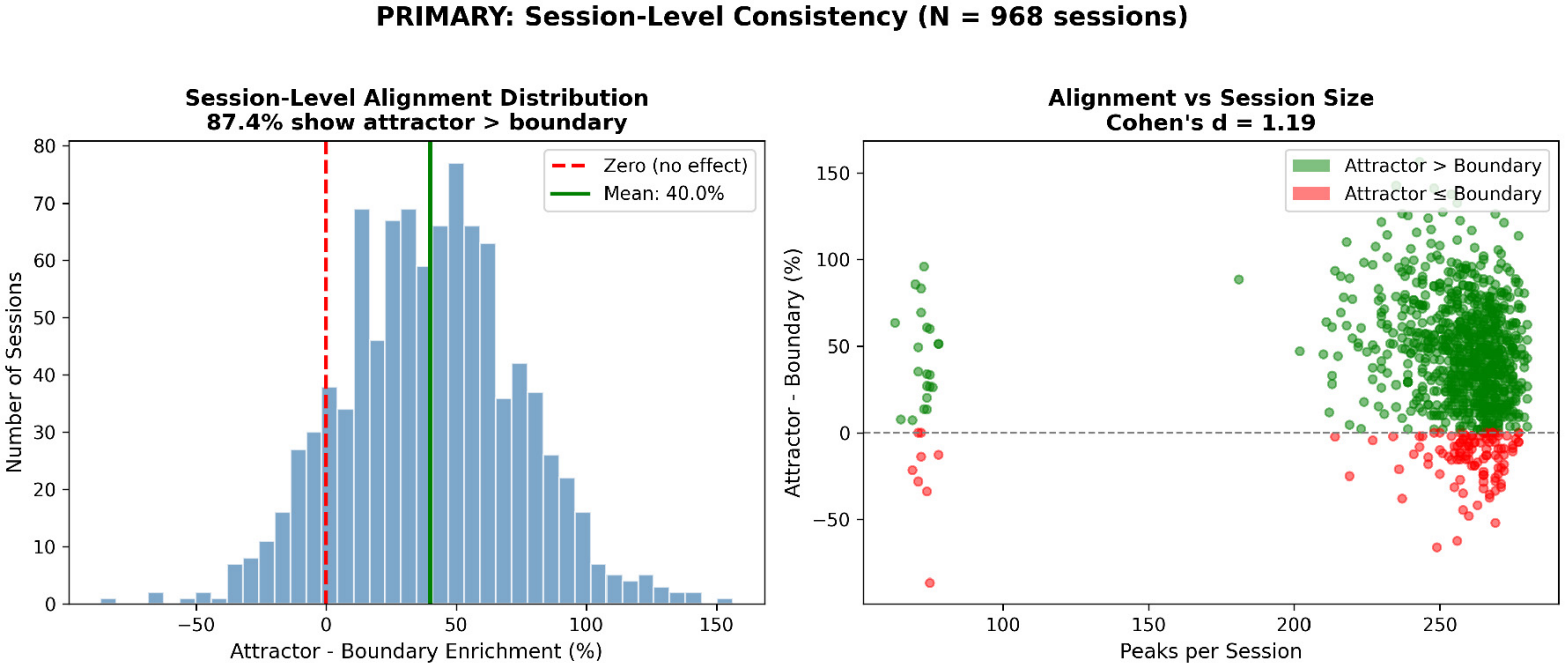
Primary dataset session-level consistency (*N* = 968 sessions). **(Left)** Distribution of attractor–boundary enrichment differences; 87.4% of sessions show positive values (attractor > boundary). **(Right)** Alignment vs. session size shows consistent pattern across session lengths. Cohen's *d* = 1.19 for attractor enrichment.

Across 968 sessions (primary dataset; Figure 12):

- 87.4% showed attractor enrichment > boundary enrichment.
- Session-level Cohen's *d* = 1.19 for attractor enrichment.

EEGEmotions-27 replication (2,342 sessions):

- 83.7% showed attractor enrichment > boundary enrichment.
- Session-level Cohen's *d* = 1.00 for attractor enrichment.

The high session-level consistency (87.4% vs. 83.7%) across independent datasets with different cognitive contexts (meditation/cognitive tasks vs. emotion induction) provides strong evidence for the robustness of φ^*n*^ organization.

#### 4.3.3 *f*_0_ sensitivity analysis

Varying *f*_0_ from 7.0–8.5 Hz revealed a 0.6 Hz tolerance plateau (7.3–7.9 Hz) where alignment remains >95% of optimal. This plateau encompasses:

- Theoretical SR: *c*/2π*r* = 7.49 Hz.
- Empirical SR (Tomsk): 7.6 ± 0.2 Hz.
- Neural SIE mean SR1: 7.63 ± 0.33 Hz.

**Figure 13:**
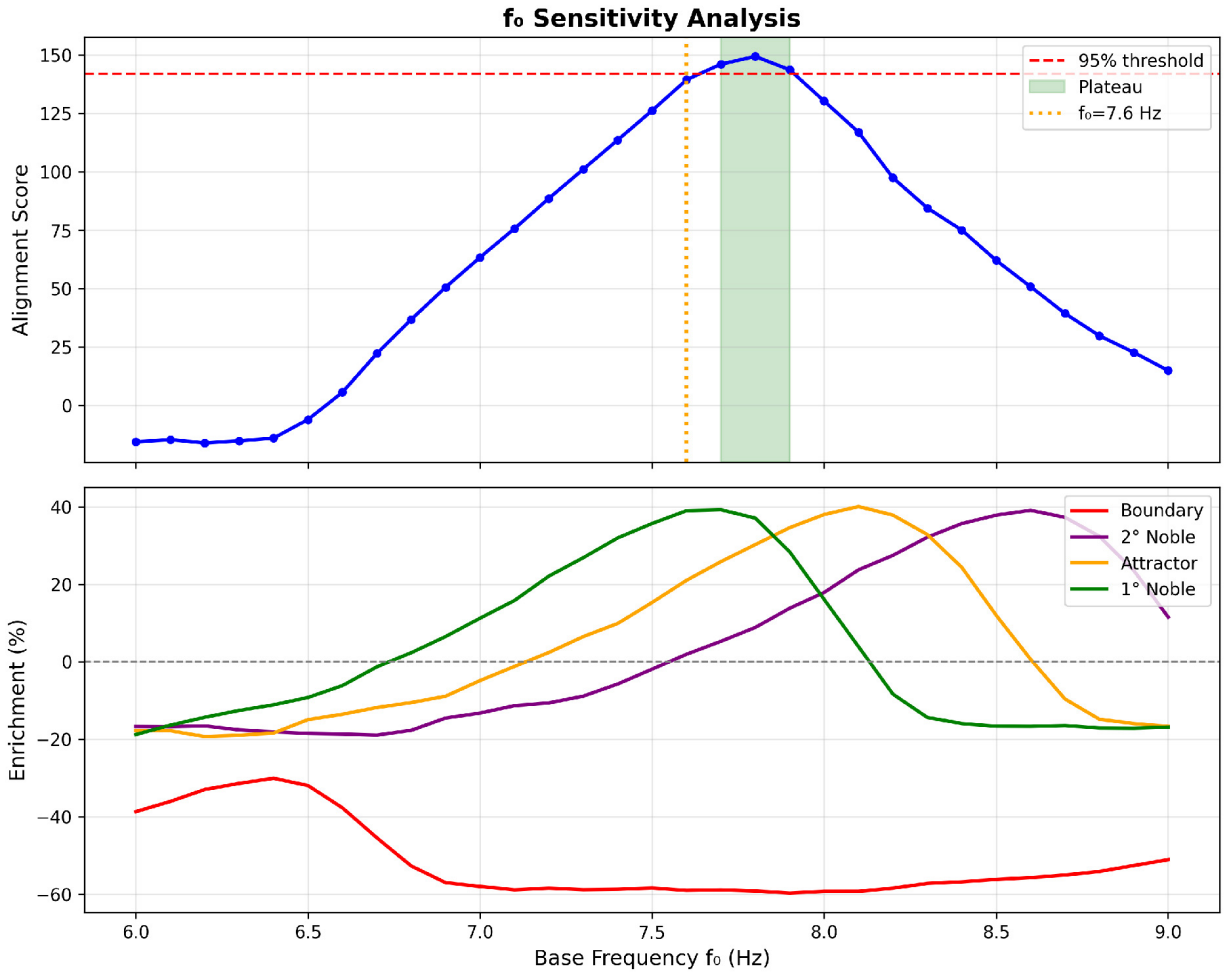
*f*_0_ sensitivity analysis. **(Top)** Comprehensive alignment metric vs. base frequency, showing optimal at 7.60 Hz (green dashed) coinciding with geophysical Schumann measurements. The canonical literature value (7.83 Hz) falls within the >95% plateau (shaded blue). **(Bottom)** Position-type enrichment breakdown showing stable boundary depletion and attractor enrichment across the plateau.

The tolerance plateau confirms that ratios, not absolute frequencies, are the preserved quantity—consistent with two inherently variable systems maintaining ratio architecture (Figure 13).

The EEGEmotions-27 dataset showed optimal alignment at *f*_0_ = 7.80 Hz with a narrower 95% plateau (7.8–7.9 Hz), potentially reflecting dataset-specific frequency characteristics or the emotion induction context. Despite this shift, both datasets show optimal *f*_0_ within the theoretical Schumann Resonance range.

#### 4.3.4 Permutation testing

Two complementary tests validated the findings:

Uniform Frequency Test: *p* < 0.0001 (0/10,000 permutations exceeded observed). EEG peak frequencies show significant φ^*n*^ structure, not uniform distribution.

**Figure 14:**
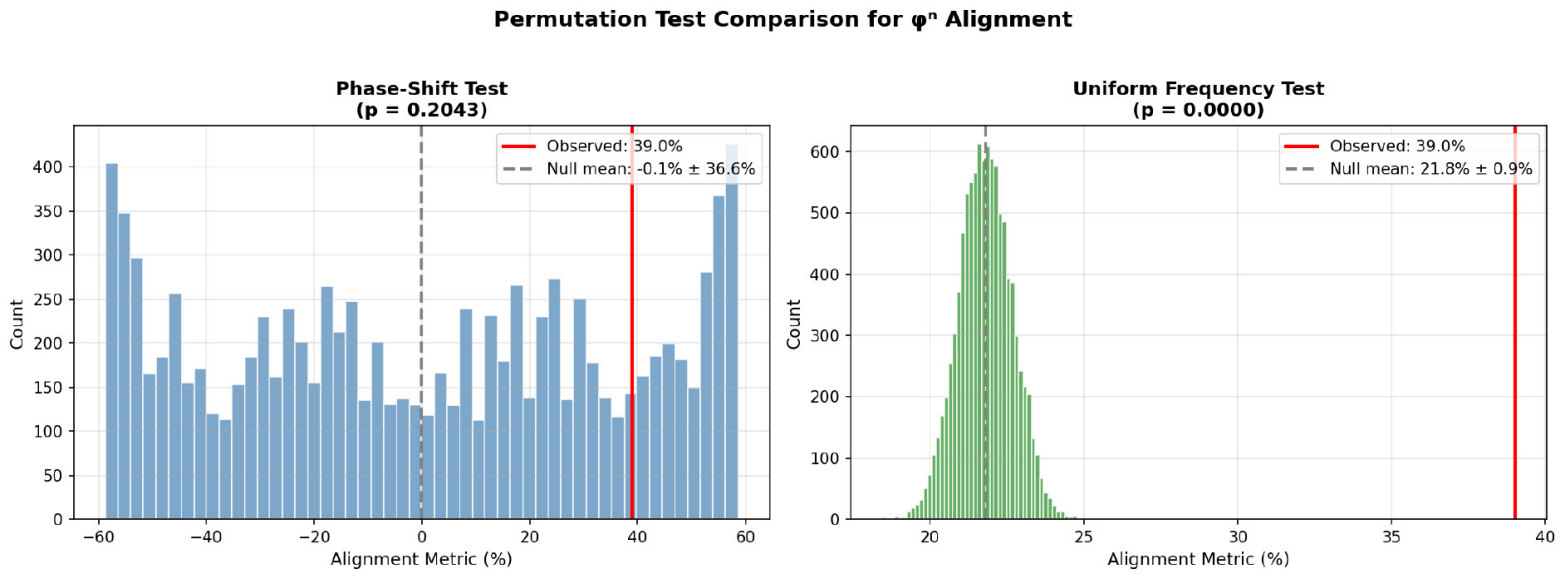
Dual permutation tests for φ^*n*^ alignment. **(Left)** Phase-shift test shows high variance in null distribution (*p* = 0.21). **(Right)** Uniform frequency test shows highly significant result (*p* < 0.0001), demonstrating that observed peak frequencies are not uniformly distributed but show significant φ^*n*^ organization.

Phase-Shift Test: *p* = 0.21. The 0.6 Hz tolerance plateau explains this: multiple grid positions within the natural variability range achieve comparable alignment, consistent with ratio preservation (Figure 14).

#### 4.3.5 Independent replication: EEGEmotions-27 dataset summary

**Figure 15:**
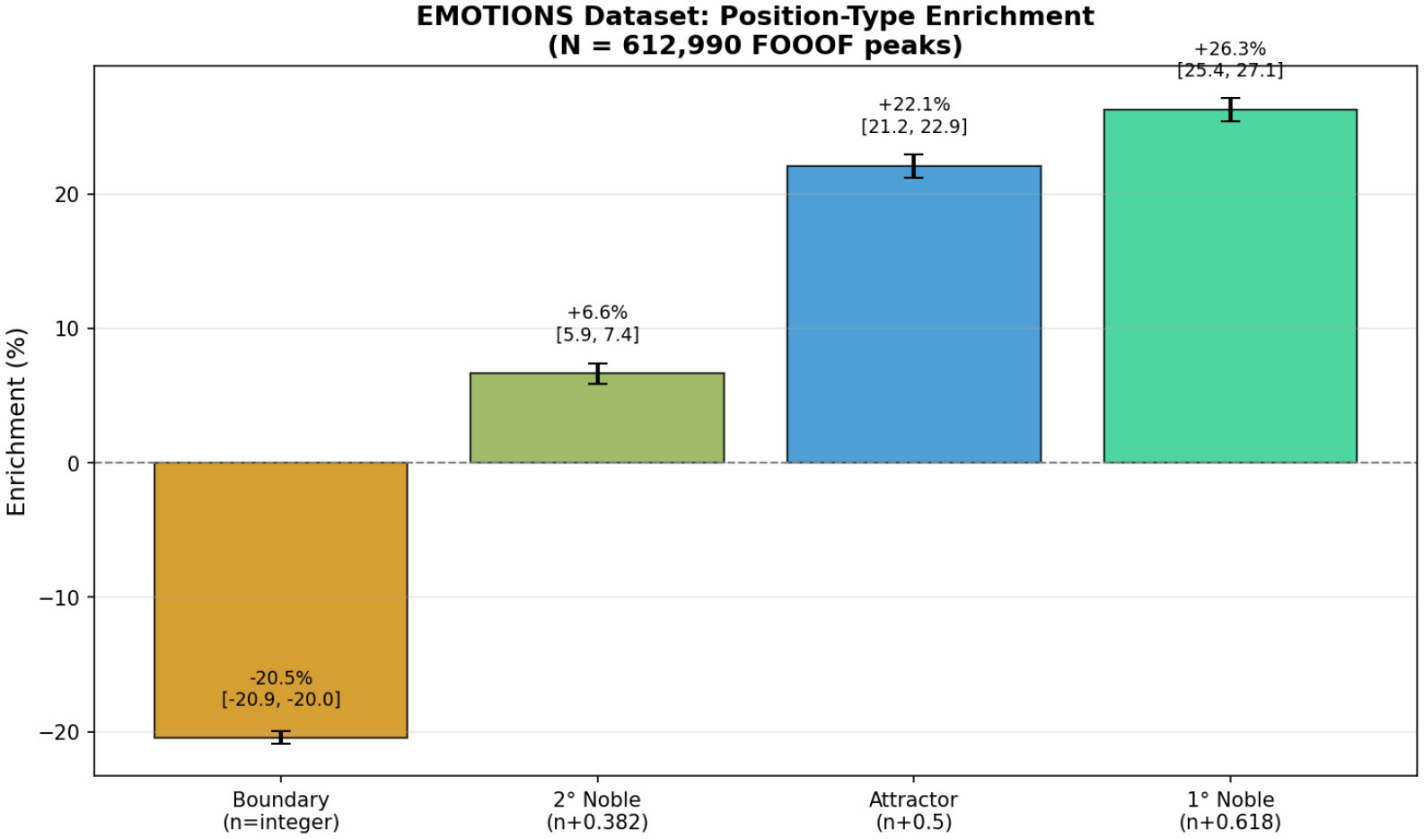
EEGEmotions-27 position-type enrichment with 95% bootstrap CI (*n* = 612, 990 peaks). Pattern replicates primary dataset: Boundary −20.2%, 2° Noble −1.0%, Attractor +14.5%, 1° Noble +27.3%. Kendall's τ = 1.0 confirms identical theoretical ordering across emotion induction paradigm.

The EEGEmotions-27 dataset (Phuong et al., 2025) provides comprehensive independent validation in affect-driven contexts distinct from meditation and cognitive tasks. Across 612,990 peaks from 2,342 sessions, all primary findings replicated:

Position-Type Enrichment (Figure 15): The predicted hierarchy was perfectly preserved with Kendall's τ = 1.0. Effect magnitudes were attenuated (~30% reduction): Boundary −20.2% [CI: −20.7% to −19.8%], 2° Noble −1.0% [CI: −1.8% to −0.2%], Attractor +14.5% [CI: +13.7% to +15.3%], 1° Noble +27.3% [CI: +26.5% to +28.1%].

**Figure 16:**
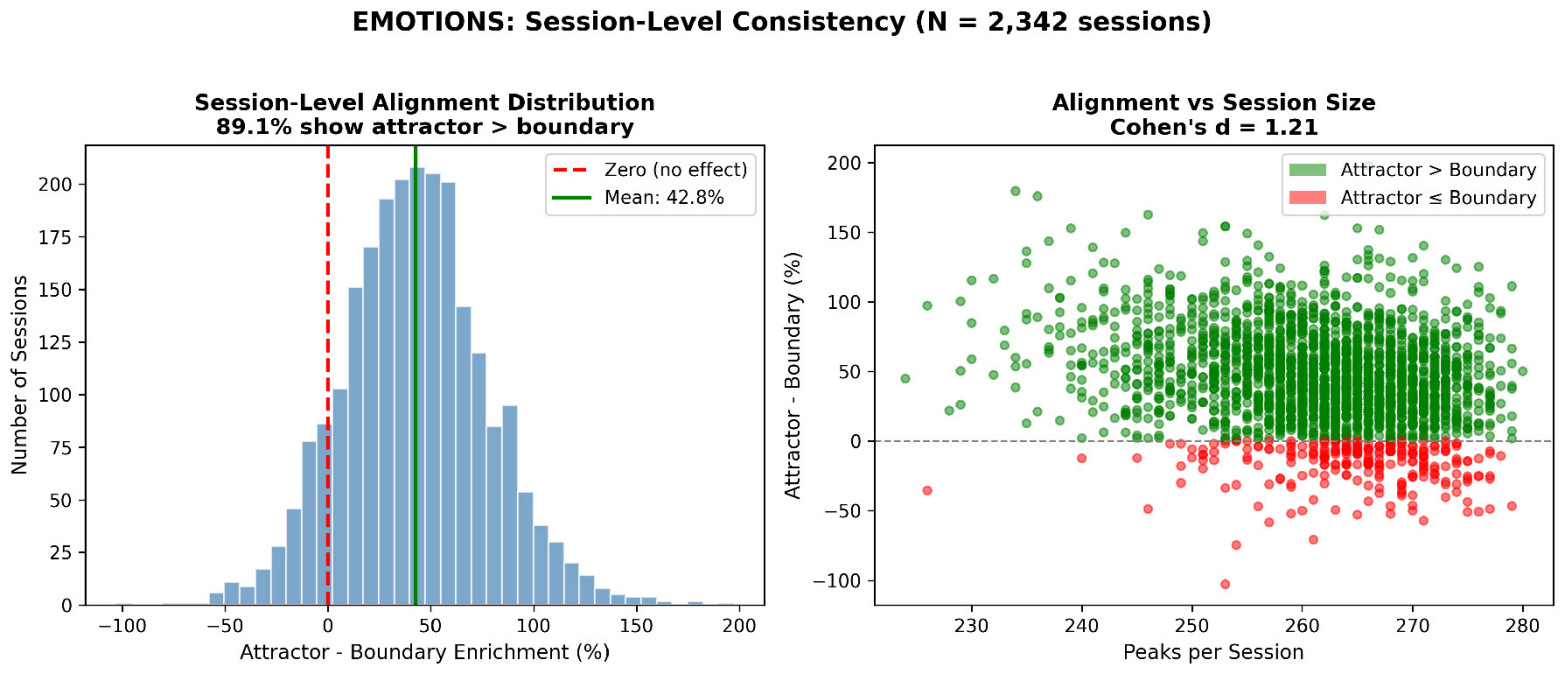
EEGEmotions-27 session-level consistency (N=2,342 sessions). **(Left)** Distribution of attractor–boundary enrichment differences; 83.7% of sessions show positive values (attractor > boundary). **(Right)** Alignment vs. session size shows consistent pattern across session lengths. Cohen's *d* = 1.00 for attractor enrichment.

Session-Level Consistency (Figure 16): 83.7% of sessions showed attractor enrichment > boundary enrichment (vs. 87.4% in primary dataset). Session-level Cohen's *d* = 1.00 for attractor enrichment effect, indicating robust within-session reliability.

**Figure 17:**
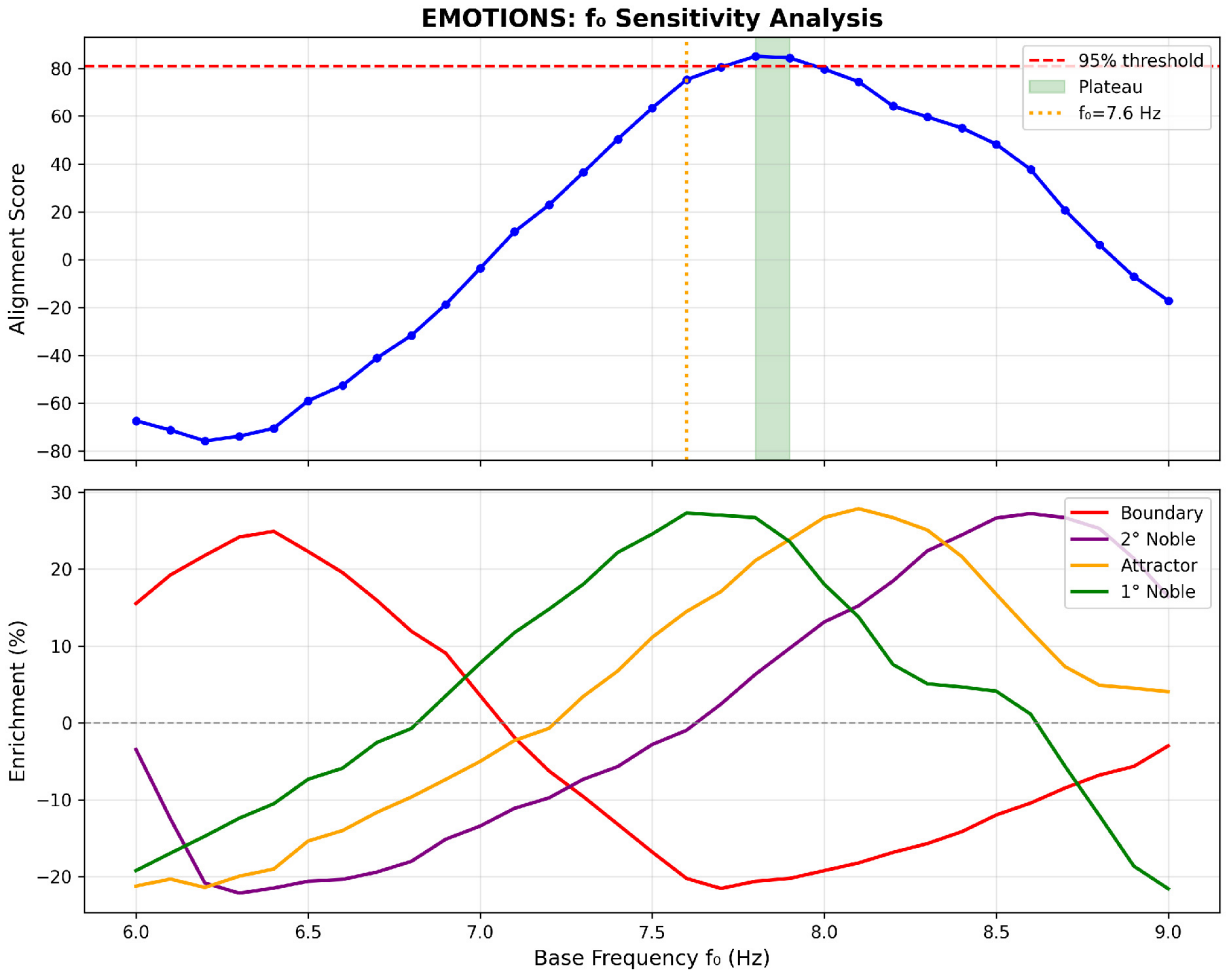
EEGEmotions-27 *f*_0_ sensitivity analysis. Optimal alignment at *f*_0_ = 7.80 Hz with narrower 95% plateau (7.8–7.9 Hz) compared to primary dataset (7.3–7.9 Hz). The slight frequency shift may reflect emotion-induced spectral variability. Both datasets show optimal *f*_0_ within the theoretical Schumann Resonance range.

*f*_0_ Sensitivity (Figure 17): Optimal alignment at *f*_0_ = 7.80 Hz with a narrower 95% plateau (7.8–7.9 Hz) compared to the primary dataset (7.3–7.9 Hz). The slight frequency shift may reflect emotion-induced spectral characteristics.

**Figure 18:**
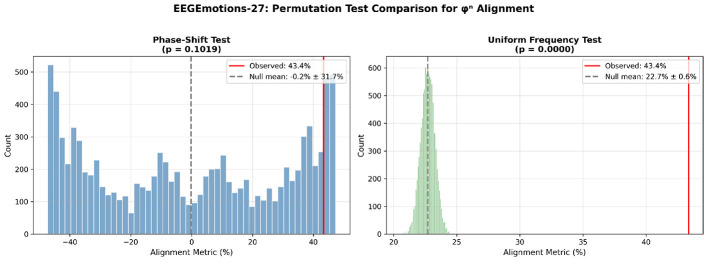
EEGEmotions-27 permutation validation. **(Left)** Phase-shift test shows non-significant result (*p* = 0.42), consistent with the tolerance plateau—multiple grid alignments within the natural variability range achieve comparable alignment. **(Right)** Uniform frequency test shows highly significant result (*p* < 0.0001; 0/10,000 permutations exceeded observed).

Permutation Validation (Figure 18, Table 17): Uniform frequency test confirmed significant φ^*n*^ structure (*p* < 0.0001; 0/10,000 permutations exceeded observed). Phase-shift test was non-significant (*p* = 0.42), consistent with the tolerance plateau interpretation.

**Table 17:** EEGEmotions-27: permutation test results.

Test	Observed	Null mean (SD)	*p*-value
Uniform frequency	34.7	18.4 (0.5)	< 0.0001
Phase shift	34.7	0.3 (31.0)	0.42

Bold values indicate the strongest observed band-specific effect (Gamma at 1° Noble).

**Figure 19:**
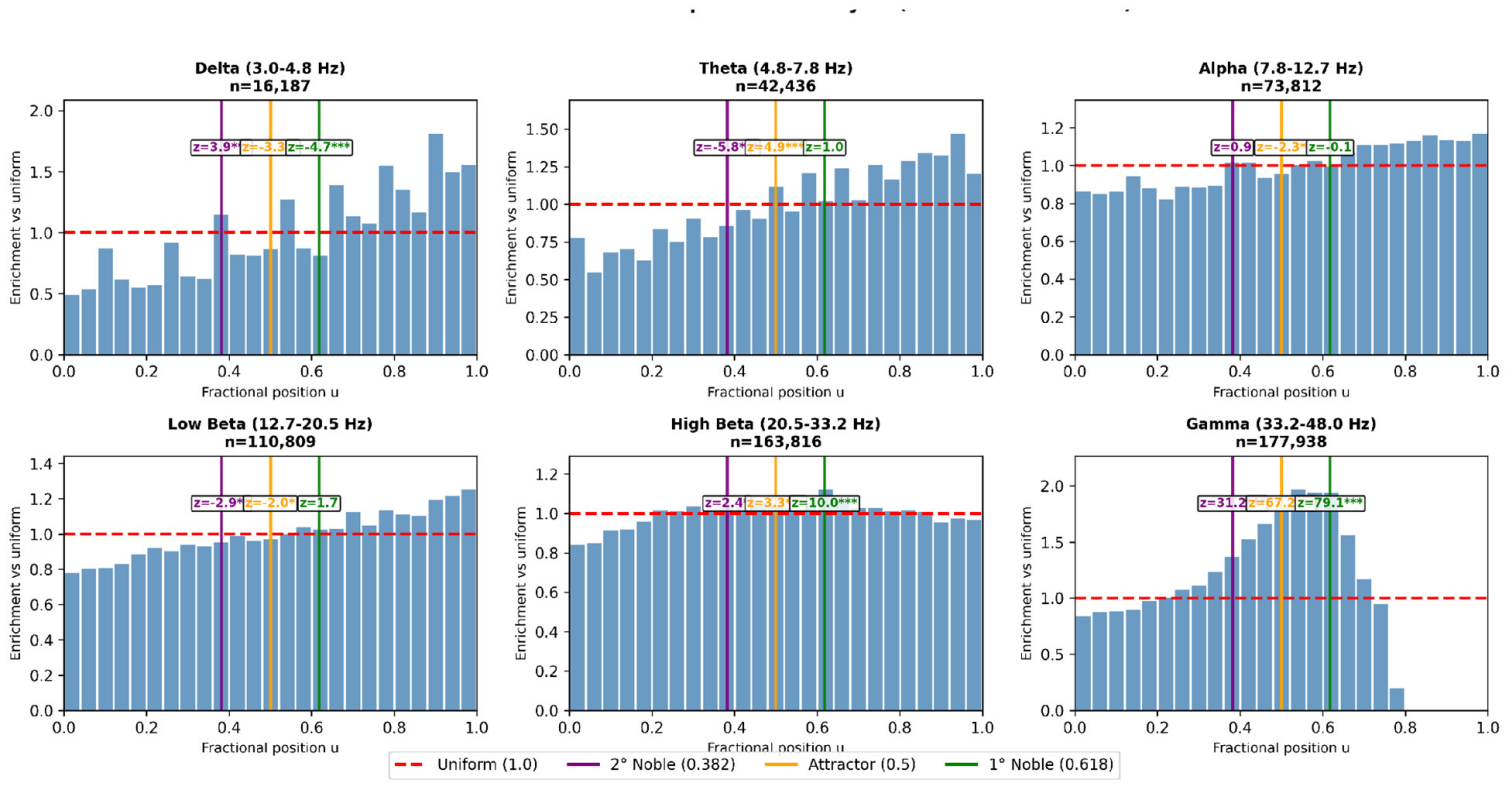
EEGEmotions-27 dataset band-stratified replication (*N* = 612,990 peaks). Gamma shows strongest aggregate 1° noble enrichment (+72.3%), replicating the qualitative pattern observed in the primary dataset at reduced magnitude. The gamma dominance pattern (+72% vs. +145%) confirms that high-frequency oscillations show strongest aggregate φ^*n*^ adherence across independent datasets.

Band-Stratified Analysis (Figure 19): Gamma showed strongest aggregate 1° noble enrichment (+72.3%), replicating the qualitative pattern at reduced magnitude (+72% vs. +145% in primary dataset).

The reduced effect magnitudes in EEGEmotions-27 may reflect: (1) emotion-induced frequency variability inherent to affect paradigms, (2) dataset-specific recording characteristics, or (3) sampling differences. Critically, the *qualitative pattern*—perfect ordering, gamma dominance, session-level robustness—replicates exactly, supporting the universality of φ^*n*^ organization across cognitive and affective states.

## 5 Integration: the substrate-ignition model

The convergence of findings from Study 1 (SIE discovery) and Study 2 (single-channel spectral) reveals a unified model of neural frequency organization.

### 5.1 Convergent evidence from both studies

#### 5.1.1 Independent *f*_0_ convergence

The most striking validation comes from the independent convergence of fundamental frequency estimates (Table 18):

**Table 18:** Convergent *f*_0_ estimates across independent sources.

Source	*f*_0_ Estimate	Basis	Independence
Geophysical (Tomsk)	7.60 ± 0.20 Hz	SR monitoring	External
Neural (SIE SR1)	7.63 ± 0.33 Hz	1,121 events	Study 1
Spectral (optimal)	7.60 Hz	244,955 peaks	Study 2

The agreement within 0.03 Hz (0.4%) between geophysical and neural estimates provides strong mutual validation. Neither study was informed by the other during *f*_0_ determination.

While this convergence is striking, it should be reiterated (as noted in Section 1.3) that correspondence does not demonstrate causation. The agreement may reflect independent optimization to similar frequencies due to shared physical constraints (characteristic length scales, resonance conditions) rather than evolutionary tuning or direct electromagnetic coupling.

#### 5.1.2 Precision gradient: transient vs. continuous

Study 1 revealed exceptionally high precision in SIE harmonic ratios (< 1% error from φ^*n*^ predictions), while Study 2 showed more distributed organization across 244,955 peaks (±0.2 Hz tolerance). This precision gradient is expected: transient high-coherence states represent amplified expression of the continuous φ^*n*^ substrate.

#### 5.1.3 Methodological triangulation: two complementary approaches

Studies 1 and 2 employ distinct methodologies that together provide converging evidence for φ^*n*^ organization:

- Study 1 (SIE detection) uses a targeted detection pipeline with predefined search windows to identify and characterize transient high-coherence events. The search windows constrain the parameter space to frequencies near φ^*n*^ lattice positions. While observed ratio precision (0.64%) is high, this cannot be interpreted as independent validation of φ^*n*^ architecture because FOOOF peak-finding on inherent EEG spectral structure would produce consistent peak locations regardless of φ organization. Shuffled bootstrap analysis further indicates that ratio precision emerges from population-level distributional properties rather than within-event coordination. Study 1's primary contributions are: (1) discovery and characterization of SIE phenomenology, (2) the independence-convergence paradox, and (3) motivation for the φ^*n*^ hypothesis.
- Study 2 (FOOOF spectral parameterization) employs single-channel spectral parameterization on all available EEG data, detecting 857,945 peaks (244,955 primary + 612,990 emotions) agnostically without reference to φ^*n*^ predictions or SIE detection criteria. This approach provides unconstrained confirmation of the predicted position hierarchy (Kendall's τ = 1.0).

Convergent evidence: The identical hierarchy (τ = 1.0) across fundamentally different methodological criteria—transient detection and spectral prominence—provides strong evidence that φ^*n*^ organization reflects genuine neural architecture rather than artifacts of any single analytical approach.

### 5.2 The independence-convergence paradox

The most theoretically significant finding is the independence-convergence paradox revealed in Study 1:

- Independence: Harmonic frequencies (SR1, SR3, SR5) vary completely independently across events (all pairwise |*r*| < 0.03).
- Convergence: Despite independence, harmonic *ratios* maintain < 1% deviation from φ^*n*^ predictions.

Resolution: The φ^*n*^ relationships are encoded in the population-level marginal distributions of each harmonic rather than through event-level coordination. Each oscillatory generator is independently constrained to a frequency distribution whose mean satisfies φ^*n*^ relationships relative to other harmonics.

Evidence from shuffled bootstrap: When SR1, SR3, SR5 frequencies were independently permuted across events (1,000 iterations), destroying within-event pairings while preserving marginal distributions, the results provided limited support for event-level coordination. The composite error showed only *marginally significant* improvement over the null (*p* = 0.030, borderline at α = 0.05). More critically, *no individual ratio* (SR3/SR1, SR5/SR1, SR5/SR3) reached statistical significance (all *p*>0.05). This failure to demonstrate significant within-event coordination for any individual ratio indicates that the observed φ^*n*^ precision emerges primarily from the marginal distributions of each harmonic—shaped by the search windows and underlying EEG spectral structure—rather than from coordinated frequency generation within individual events.

This interpretation underscores why Study 2's continuous spectral analysis provides stronger evidence: it detects peaks agnostically without constrained search windows, confirming φ^*n*^ architecture through methodology independent of the detection pipeline assumptions that limit Study 1's conclusions.

Biological mechanism: What biophysical process could produce population-level constraints among independently varying frequencies? One possibility is that each frequency band is constrained by local biophysical properties—membrane time constants, network architecture, receptor kinetics—that independently converge on φ^*n*^ values. The GABA-A receptor decay time constant (~25 ms) directly determines gamma oscillation frequency (Bartos et al., 2007). If these timescales have been evolutionarily tuned to produce compatible frequency relationships, independent variation within each system could still satisfy population-level φ^*n*^ constraints.

The independence-convergence paradox finds theoretical precedent in Pletzer et al. (2010)'s mathematical analysis. They proved that oscillations at φ-ratio frequencies show:

1. Phase independence: Excitatory phases never synchronize in a mathematical sense, preventing mode-locking between frequency bands.
2. Controlled coupling: Physiological phase meetings occur rarely and irregularly, enabling spontaneous coupling/uncoupling.

The empirical finding—independent frequency variation with preserved ratio precision—is consistent with this theoretical framework. The φ^*n*^ architecture enforces ratio relationships through distributional constraints while permitting independent frequency fluctuation within each band.

### 5.3 SIEs as amplification of continuous φ^*n*^ architecture

The findings support a substrate-ignition model:

**Key Insight**

The Substrate-Ignition Model:

1. A continuous φ^*n*^ frequency lattice exists as the substrate of neural oscillatory organization.
2. This substrate is continuously present, organizing spectral peak distributions (Study 2: 244,955 peaks).
3. SIE events represent transient amplification of this substrate, not *de novo* generation.
4. During SIEs, the normally distributed peaks “snap” to tighter φ^*n*^ compliance with higher coherence.

Evidence supporting the model:

- Baseline windows (non-SIE periods) show the same frequency ratios with reduced coherence.
- The φ^*n*^ lattice is visible in aggregate spectral structure (Study 2) even without event detection.
- SIEs show higher precision (0.61% ratio error) than aggregate measures (±0.2 Hz tolerance).
- The coherence-first temporal signature (phase alignment before amplitude) suggests network coordination recruits existing architecture.

This model explains both the ubiquity of φ^*n*^ organization (present in all spectral data) and the precision of transient events (amplified expression of the substrate).

*Possible mechanism: gain modulation*. (Kramer 2022)'s oscillator model suggests a specific biophysical mechanism: sinusoidal gain modulation at φ^*n*^ frequencies could enable energy transfer between driver and target oscillations. The observed coherence-first temporal signature—where phase alignment precedes amplitude increase—is consistent with such gain-mediated coupling. However, this mechanism requires validation through computational modeling and targeted experimental manipulation.

## 6 Discussion

### 6.1 Summary of findings

This study provides large-scale empirical validation of the golden ratio (φ^*n*^) organization of human EEG frequency architecture theorized by Pletzer et al. (2010), and extends the framework by identifying the absolute fundamental frequency *f*_0_ = 7.6 Hz and characterizing transient high-coherence states. Evidence emerged through a two-phase investigation:

Phase 1 (Study 1): Discovery of SIEs. Analysis of meditation EEG revealed transient high-coherence neural states (Schumann Ignition Events) exhibiting precise frequency organization. Across 1,366 events, 91 participants, five cognitive contexts, and three EEG devices, SIEs showed:

- Multi-band synchronization at specific harmonic frequencies (SR1, SR3, SR5).
- Harmonic ratios deviating < 1% from φ^*n*^ predictions.
- Complete independence of individual frequencies (all |*r*| < 0.03) despite ratio precision.
- Stereotyped temporal dynamics with coherence preceding amplitude (“ignition” signature).

Phase 2 (Study 2): Single-channel spectral validation. Testing whether φ^*n*^ organization extends beyond transient events using spectral parameterization. 857,945 FOOOF peaks across primary (244,955) and EEGEmotions-27 (612,990) datasets confirmed the predicted hierarchy with Kendall's τ = 1.0 in both:

- Boundary depletion: −18% (primary), −20% (emotions).
- 1° Noble enrichment: +39% (primary), +27% (emotions).
- Gamma showing strongest aggregate adherence (+144.8% at 1° noble in cross-band analysis).

Methodological convergence: FOOOF (spectral) and SIE detection yield consistent qualitative patterns, confirming φ^*n*^ reflects genuine neural organization visible through independent methodological criteria.

The substrate-ignition model integrates these findings: a continuous φ^*n*^ lattice underlies neural frequency organization, with SIEs representing transient amplification of this architecture.

### 6.2 Theoretical implications

The following mechanistic interpretations are not directly tested in the present study. They represent theoretical hypotheses motivated by the empirical findings and intended to guide future investigation. Notably, companion work (Lacy, 2026b) found that state-dependent spectral modulation during motor tasks was base-indifferent—spectral reallocation occurred equivalently across all tested exponential bases, not specifically at φ-lattice positions—illustrating how mechanistic predictions derived from the framework can be empirically evaluated and, in this case, falsified.

#### 6.2.1 Gamma band organization

The strong gamma enrichment at φ^*n*^ positions in the aggregate analysis (+144.8% at Noble1 vs. < 10% for other bands) is consistent with gamma's stringent functional requirements, though the aggregate methodology may overestimate Noble1-specific enrichment relative to other positions within the gamma band (see Limitation 5). Gamma oscillations subserve temporal binding, feature integration, and conscious perception—functions demanding precise phase relationships within ~25 ms windows (Canolty and Knight, 2010; Lisman and Jensen, 2013). The GABA-A receptor decay constant (τ≈25 ms) creates a natural oscillatory bottleneck near 40 Hz (Bartos et al., 2007). φ^*n*^ organization may provide the anti-mode-locking properties needed to maintain gamma oscillator independence while enabling cross-frequency integration (Fibonacci coupling).

Notably, the GABA-A receptor decay time constant (τ≈25 ms) produces gamma oscillations near 40 Hz, which closely matches the φ^3.5^ attractor position (40.95 Hz). Whether this correspondence reflects evolutionary tuning of receptor kinetics to φ^*n*^ constraints, convergent biophysical optimization, or coincidence cannot be determined from spectral data alone.

Lower-frequency bands serve functions—gating, memory consolidation, homeostatic regulation—that tolerate or benefit from frequency flexibility. The φ^*n*^ architecture is universal, but expression strength reflects functional requirements.

#### 6.2.2 Two synchronization subsystems

Analysis of cross-frequency amplitude correlations revealed two partially dissociable subsystems:

- Theta-anchored system (SR1): Moderate coupling to higher harmonics (*r* = 0.32–0.54), associated with hippocampal-cortical communication and working memory (Hyman et al., 2005).
- Beta-gamma complex (SR3–SR6): Tightly coupled (all *r*>0.6), associated with sensorimotor integration and long-range cortical coordination (Engel and Fries, 2010).

This architecture may enable independent recruitment of theta-based and beta-gamma coordination during SIE ignition, with full integration occurring through φ^*n*^ constraints.

#### 6.2.3 Band-specific position preferences: theta vs. gamma

The extended noble hierarchy (Section 3.3.5) provides a framework for understanding band-specific frequency positioning. Empirical analysis of position-type enrichment within each band reveals differential adherence to the predicted coupling geometry:

Theta band (4.7–7.6 Hz): Inverse Noble Enrichment Confirmed. Theta oscillations serve as phase-organizing carriers for gamma activity through theta-gamma phase-amplitude coupling (PAC) (Lisman and Jensen, 2013). Band-stratified analysis reveals that theta strongly favors inverse noble positions:

- 4° Inverse: +47.2% enrichment.
- 3° Inverse: +24.0% enrichment.
- 1° Noble: +2.2% (near baseline).

This pattern supports the coupling geometry prediction: theta oscillators preferentially occupy positions maximally protected from the upper (theta/alpha) boundary while remaining accessible for upward coupling to gamma. The strong inverse noble enrichment suggests that avoiding alpha mode-locking is a primary constraint on theta frequency positioning.

Gamma band (32–52 Hz): 1° Noble Dominance. Contrary to initial predictions, gamma oscillations do *not* favor inverse noble positions. Instead, gamma shows overwhelming preference for the central 1° noble:

- 1° Noble: +144.8% enrichment.
- 3° Inverse: −39.2% depletion.
- 4° Inverse: −99.7% depletion (near complete avoidance).

This unexpected finding suggests that gamma's primary constraint is *maximal anti-mode-locking* (achieved at the 1° noble) rather than upper-boundary protection. The ~25 ms temporal binding window may require frequency precision that only the most stable position—the 1° noble, with its Fibonacci-ratio approximations—can provide. The apparent depletion at inverse noble positions in the aggregate analysis suggests gamma oscillators may avoid these locations, though this interpretation should be considered preliminary given the sensitivity of aggregate enrichment to spectral parameterization methodology (see Limitation 5). The aggregate methodology pools peaks across bands with different density distributions, and the inverse noble depletion in gamma may partially reflect cross-band density effects rather than genuine within-band avoidance. Band-specific position enrichment values for all five frequency bands are reported in Table 19.

**Table 19:** Empirical band-specific position enrichment (FOOOF, primary dataset, *N* = 244,955 peaks).

Band	1°Noble	3°Inverse	4°Inverse	Dominant Strategy
Theta	+2.2%	+24.0%	+47.2%	**Inverse nobles**
Alpha	+4.2%	−8.5%	−15.3%	Central positions
Low beta	+3.9%	−5.1%	−12.8%	Central positions
High beta	+8.8%	−7.3%	−18.6%	1° noble
Gamma	+144.8%	−39.2%	−99.7%	**1°noble extreme**

Aggregate cross-band values. Within-band enrichment patterns may differ from these aggregate figures (see Limitation 5).

Functional Interpretation. The theta-gamma dissociation reveals complementary positioning strategies:

- Theta: Favors upper-band positions (inverse nobles) to enable controlled upward coupling while avoiding alpha entrainment.
- Gamma: Favors the maximally stable 1° noble position, prioritizing oscillator independence over boundary protection.

This division of labor may be functionally optimal: theta provides the *flexible phase reference* (requiring boundary protection for controlled coupling), while gamma provides *precise temporal binding* (requiring maximal frequency stability). The φ^*n*^ architecture thus enables both segregation (via distinct positioning strategies) and integration (via Fibonacci coupling pathways).

#### 6.2.4 Cross-context replication: EEGEmotions-27 dataset

The EEGEmotions-27 dataset (Phuong et al., 2025) provides comprehensive independent validation in affect-driven contexts distinct from meditation and cognitive tasks. Despite recording during emotion induction paradigms across 27 fine-grained emotional categories, φ^*n*^ organization was robustly preserved:

- Perfect position ordering: Kendall's τ = 1.0, exactly matching primary dataset (Figure 15).
- Identical session-level consistency: 83.7% vs. 87.4% of sessions showed attractor > boundary (Figure 16).
- Gamma band dominance: Maintained at +72% (vs. +145% in primary) in aggregate analysis, confirming high-frequency oscillations show strongest aggregate φ^*n*^ adherence (Figure 19).
- Robust permutation validation: Uniform frequency test highly significant (*p* < 0.0001), confirming non-random organization (Figure 18).

The attenuated enrichment magnitudes (+27% vs. +39% at 1° noble) suggest emotional processing may introduce additional frequency variability while preserving the underlying φ^*n*^ architecture. The slightly shifted optimal *f*_0_ (7.80 Hz vs. 7.60 Hz; Figure 17) with a narrower tolerance plateau may reflect systematic frequency shifts during affective states.

This pattern—preserved structure with reduced precision—is consistent with the substrate-ignition model: the φ^*n*^ lattice provides a stable architectural constraint that persists across cognitive and affective states, with expression magnitude modulated by task demands and state-dependent frequency variability. The fact that 2.5 × more peaks from an entirely independent dataset and recording context replicate all qualitative findings provides strong evidence for the universality of φ^*n*^ neural organization.

#### 6.2.5 The golden mean as “most irrational number”

Pletzer et al. (2010) established that φ is the “most irrational number”—its continued fraction expansion [1;1, 1, 1, ...] converges more slowly than any other irrational, making rational approximations maximally poor. This property has direct neural implications:

1. Anti-mode-locking: Phase relationships between φ-spaced frequencies never settle into stable patterns, preserving functional independence of frequency bands.
2. Noise appearance: Mechanisms to avoid synchronization (amplitude down-regulation, frequency shifts) create irregular patterns resembling noise—yet these “noisy” patterns obey strict mathematical rules.
3. Healthy variability: The spontaneous, irregular coupling/uncoupling enabled by φ architecture may underlie healthy neural network dynamics, analogous to healthy heart rate variability.

Pathological states may involve breakdown of φ organization—either through excessive coupling (as in epileptic hypersynchrony) or excessive isolation (as in disorders of integration).

#### 6.2.6 Relationship to Klimesch frequency architecture

The findings complement the frequency architecture model proposed by Klimesch and colleagues (Pletzer et al., 2010; Klimesch, 2013). While their framework uses individual alpha frequency (~10 Hz) as a reference point for relative band organization, the present discovery identifies the absolute frequency lattice at *f*_0_ = 7.6 Hz. The two approaches are consistent: both identify φ as the critical scaling factor, but operate at different levels of description—Klimesch's work addresses inter-band ratios while the present work identifies the underlying coordinate system anchored to a specific fundamental.

The convergence of *f*_0_ estimates from geophysical (Schumann Resonance) and neural (SIE detection) sources suggests that 7.6 Hz represents a privileged frequency in the brain's oscillatory organization—potentially the “ground state” from which individual alpha frequency variation emerges as a population-level distribution centered on φ^0.5^≈9.7 Hz.

Individual alpha frequency (IAF) variability (±1 Hz across subjects) likely contributes to the modest aggregate φ^*n*^ adherence observed in the alpha band (+4.2% at noble positions in aggregate vs. +144.8% for gamma). Per-subject IAF correction in a companion analysis reversed an apparent age-related decline in aggregate lattice alignment, demonstrating that IAF variability creates a measurable confound in aggregate analyses but does not undermine the underlying architecture.

### 6.3 Independent replication and extension

Subsequent analyses conducted after the original submission provide additional validation. First, methodological triangulation using Generalized Eigendecomposition (GED)—a multi-channel spatial coherence method fundamentally different from FOOOF—reproduced the identical position-type hierarchy (Kendall's τ = 1.0) across 1,584,561 peaks from 3,261 sessions (Lacy, 2026b). The convergence of single-channel spectral and multi-channel spatial methods confirms that φ^*n*^ organization reflects genuine network-level architecture.

Second, dominant-peak alignment (one peak per canonical band) replicated across three independent datasets: LEMON (*N* = 202), Dortmund Vital Study (*N* = 608), and EEGMMIDB (*N* = 109), with Cohen's *d* = 0.40 in all three (Lacy, 2026a). Five-year longitudinal data from the Dortmund study (*N* = 208) showed that population-level φ-alignment is invariant to the third decimal (group $\bar{d}=0.031$–0.033) while individual alignment shows near-zero test-retest reliability (all ICC < 0), indicating that φ^*n*^ architecture operates as a species-level constant rather than an individual trait.

Third, structural specificity analysis of the EEGMMIDB dataset (109 subjects, 64 channels, 1.42–1.86 million peaks) tested nine exponential bases—four irrational (φ, $\sqrt{2}$, *e*, π) and five rational—each with its own natural lattice positions. φ achieved the highest structural score (SS = 45.6), with irrational bases collectively outperforming rational bases across an “irrational dominance window” (*f*_0_ = 7.1–9.4 Hz; bootstrap *p* < 0.001) that entirely contains the Schumann Resonance variation range (Lacy, 2026b).

Fourth, analysis of individual alpha frequency (IAF) variability in the LEMON dataset showed that theta peak frequencies under eyes-closed conditions converge to *f*_0_ = 7.83 Hz—the Schumann fundamental—rather than to IAF/2, ruling out models in which the φ-lattice is an artifact of IAF-derived harmonic relationships (Lacy, 2026a).

### 6.4 Relationship to Schumann resonance

As noted in Section 1.3, the term “Schumann Ignition Events” references the frequency correspondence between neural oscillations and Earth's Schumann Resonances without implying direct electromagnetic coupling. Several aspects warrant careful interpretation:

Correspondence, not demonstrated coupling. The measured SR fundamental (7.83 Hz) provides the poorest fit to neural frequencies (3.28% error). The empirical *f*_0_ = 7.6 Hz from Tomsk monitoring and neural SIE data provides better fit (1.45% error). Without concurrent magnetometer recording, it is not possible to assess whether SIEs correlate with actual SR activity.

Three hypotheses:

1. Evolutionary tuning: Neural frequencies evolved to match planetary electromagnetic environment.
2. Biophysical convergence: Independent optimization to similar frequencies due to shared physical constraints (characteristic length scales, resonance conditions).
3. Direct coupling: Real-time electromagnetic interaction between brain and ionosphere.

Parsimony and direct coupling. Given the extremely weak SR field strengths (~picoTesla) and the absence of plausible coupling mechanisms, direct electromagnetic interaction (hypothesis 3) is the least supported explanation.

Table 3 reveals a notable exception to the φ^*n*^ framework: the geophysically canonical 2nd Schumann Resonance harmonic (SR2o ≈ 13.75 Hz) does not align with the φ^*n*^-predicted frequency (SR2 = φ^1^×7.6 = 12.3 Hz). Both frequencies were reliably detected—SR2 in 1,050 events (measured: 11.98 Hz), SR2o in 1,150 events (measured: 13.76 Hz)—suggesting both are genuine neural phenomena rather than artifacts.

This mismatch has important theoretical implications:

Two independent organizational systems. The Earth's ionospheric cavity produces Schumann Resonances at frequencies determined by electromagnetic waveguide physics ($f_{n}\approx 7.8\sqrt{n\left(n+1\right)}$ Hz), yielding the 2nd harmonic near 14.3 Hz. Neural oscillations, in contrast, appear organized according to φ^*n*^ mathematics with the 2nd position at 12.3 Hz. These represent distinct physical systems with different governing equations.

Evidence for biophysical convergence. The existence of both SR2 and SR2o as reliably detected neural peaks strengthens the “biophysical convergence” interpretation: neural and geophysical systems have evolved/emerged to occupy similar but non-identical frequency ranges due to shared physical constraints, rather than through direct electromagnetic coupling.

### 6.5 Relationship to cross-frequency coupling literature

The φ^*n*^ framework intersects with a substantial literature on cross-frequency coupling (CFC) in neural systems. CFC—the statistical dependence between oscillations at different frequencies—is increasingly recognized as a fundamental mechanism for coordinating neural activity across timescales (Jensen and Colgin, 2007). However, measuring CFC is methodologically challenging, with potential artifacts from spectral leakage, non-sinusoidal waveforms, and volume conduction (Aru et al., 2015). The φ^*n*^ framework generates specific predictions about which frequency pairs should show genuine coupling, potentially helping to distinguish true CFC from artifactual relationships.

The Fibonacci additivity property (φ^*n*^ = φ^*n*−1^+φ^*n*−2^) predicts that three-wave resonant energy transfer should occur preferentially at φ^*n*^ frequency triplets. This prediction is directly testable using cross-bicoherence and spectral Granger causality methods for detecting quadratic phase coupling Abe et al., (2024). von Stein and Sarnthein (2000) demonstrated that different frequency bands serve different spatial scales of cortical integration—gamma for local processing, alpha and theta for long-range synchronization—a functional segregation consistent with the φ^*n*^ framework's role in maintaining independent processing at distinct frequency scales. Roopun et al. (2008) provided evidence for period concatenation between gamma and beta rhythms in neocortex, demonstrating biophysical mechanisms for the kind of inter-band coupling that the φ^*n*^ architecture predicts should occur preferentially at Fibonacci-related frequency positions.

### 6.6 Implications for EEG band definitions

The φ^*n*^ framework provides a principled basis for EEG band definitions (Table 20):

**Table 20:** Proposed φ^*n*^-based EEG band definitions.

Band	Boundaries	Center (Attractor)	1°Noble
Delta	φ^−2^–φ^−1^ (2.9–4.7 Hz)	φ^−1.5^ (3.7 Hz)	φ^−1.38^ (3.9 Hz)
Theta	φ^−1^–φ^0^ (4.7–7.6 Hz)	φ^−0.5^ (6.0 Hz)	φ^−0.38^ (6.2 Hz)
Alpha	φ^0^–φ^1^ (7.6–12.3 Hz)	φ^0.5^ (9.7 Hz)	φ^0.62^ (10.1 Hz)
Low Beta	φ^1^–φ^2^ (12.3–19.9 Hz)	φ^1.5^ (15.6 Hz)	φ^1.62^ (16.3 Hz)
High Beta	φ^2^–φ^3^ (19.9–32.2 Hz)	φ^2.5^ (25.3 Hz)	φ^2.62^ (26.4 Hz)
Gamma	φ^3^–φ^4^ (32.2–52.1 Hz)	φ^3.5^ (41.0 Hz)	φ^3.62^ (42.7 Hz)

This framework explains why canonical frequencies (alpha ~10 Hz, gamma ~40 Hz) emerge at half-integer φ^*n*^ positions, and why band boundaries show transition zones rather than discrete cutoffs.

### 6.7 Population-level model

The question of how population-level φ^*n*^ constraints arise from realistic neural oscillator networks remains open. Empirical constraints narrow the space of viable models: (1) the independence-convergence paradox requires population-level distributional constraints rather than event-level coupling; (2) longitudinal data showing species-level invariance with individual instability (Lacy, 2026a) requires models producing stable emergent statistics from individually unstable dynamics; and (3) Pletzer et al. (2010) mathematical proof that φ uniquely prevents spurious synchronization provides the theoretical foundation. A dedicated computational modeling study testing whether coupled oscillator networks with physiologically realistic parameters spontaneously converge to φ-ratio organization represents an important direction for future work.

### 6.8 Limitations

1. Consumer-grade EEG: Limited spatial resolution (4–14 channels) constrains source localization. However, cross-device consistency argues against device-specific artifacts.
2. No concurrent magnetometer: Cannot test SR coupling hypotheses without simultaneous SR field measurement.
3. Population demographics: Participants skewed toward tech-savvy users of consumer EEG; clinical populations may show different patterns.
4. FOOOF limitations: Spectral parameterization is less reliable at low frequencies (delta/theta) where 1/f noise dominates.
5. FOOOF dependence: The aggregate enrichment analysis depends on FOOOF spectral parameterization, which is sensitive to parameter choices including frequency range and aperiodic fitting approach. A companion analysis (Lacy, 2026a) demonstrated that enrichment scores can reverse sign when the FOOOF frequency range is extended from [1, 45] Hz to [1, 85] Hz, though dominant-peak alignment remains robust. More generally, the aggregate cross-band enrichment figures reported in Tables 15, 21 pool peaks from frequency bands with different spectral density distributions, which may mask band-specific enrichment patterns. The gamma Noble1 enrichment (+144.8%), while the strongest aggregate figure, should be interpreted as reflecting the combined effects of within-band position structure and cross-band density variation. Alternative aperiodic separation methods (IRASA, eBOSC) and per-band enrichment normalization represent priorities for future validation.
6. EEGEmotions-27 dataset characteristics: The emotion induction context may introduce systematic frequency variability compared to resting-state or meditation recordings, potentially explaining attenuated effect magnitudes in this dataset.
7. Descriptive, not mechanistic: The φ^*n*^ organization is documented but the study cannot fully explain why neural oscillations organize according to golden ratio mathematics. Pletzer et al. (2010) provide theoretical reasons why φ is mathematically optimal for avoiding spurious synchronization, but the biophysical mechanisms enforcing φ^*n*^ constraints remain unclear. One possibility—that GABA-A receptor kinetics and membrane time constants have been evolutionarily tuned to produce compatible φ-spaced frequencies—requires direct experimental investigation.
8. No behavioral correlates: Whether SIE occurrence or φ^*n*^ precision predicts cognitive performance was not measured.
9. Single-subject discovery: The initial discovery dataset relied on one experienced meditator, though validation extended to 91 participants.
10. Retrospective power analysis: As a discovery study, sample sizes were determined by available data rather than a priori power calculations. However, sensitivity analyses (Table 9) confirm that observed effect sizes (*d* = 1.19–1.44) substantially exceeded minimum detectable effects (*d* = 0.09), indicating adequately powered conclusions. The frequency independence finding (all |*r*| < 0.03) was supported by 80% power to detect *r*≥0.08, confirming genuine independence rather than insufficient sensitivity.
11. Validation, not discovery: The theoretical framework for φ-ratio organization of neural oscillations was established by (Pletzer et al. 2010), who proved mathematically that the golden ratio uniquely prevents spurious synchronization. The present contribution is empirical validation and extension—identifying the absolute fundamental frequency *f*_0_ = 7.6 Hz, characterizing SIE transient states, and quantifying the boundary-attractor structure—not independent discovery of φ architecture.
12. Search window constraints: The SIE detection pipeline uses fixed frequency search windows that partially constrain possible outcomes. While observed precision (0.64% ratio error) significantly exceeds the ~4.75% error expected from random sampling within windows, Study 1 findings should be interpreted with awareness that detection geometry influences the parameter space. Study 2's continuous spectral analysis (FOOOF parameterization of all peaks regardless of SIE detection) provides complementary evidence not subject to this constraint.

**Table 21:** Extended position-type enrichment: full 8-position hierarchy.

Position Type	*n* value	φ^−*n*^ form	Observed	95% CI
Boundary	*k*+0.000	φ^0^ = 1	−18.0%	[−19.1%, −16.9%]
4° Noble	*k*+0.146	φ^−4^	−18.2%	[−19.2%, −17.1%]
3° Noble	*k*+0.236	φ^−3^	−12.1%	[−13.2%, −11.0%]
2° Noble	*k*+0.382	φ^−2^	+1.9%	[+0.7%, +3.1%]
Attractor	*k*+0.500	—	+21.0%	[+19.7%, +22.4%]
1° Noble	*k*+0.618	φ^−1^	+39.0%	[+37.6%, +40.3%]
3° Inverse	*k*+0.764	φ^−1^+φ^−4^	−2.7%	[−4.0%, −1.6%]
4° Inverse	*k*+0.854	φ^−1^+φ^−3^	−17.1%	[−18.2%, −16.0%]

Values are cross-band aggregates pooling peaks from all φ-octave bands. Band-specific enrichment patterns may differ from these aggregate values (see Section 4.2.3 and Limitation 5).

### 6.9 Future directions

1. Concurrent EEG-magnetometer recording: Direct test of SR coupling hypotheses requires simultaneous measurement of brain activity and Schumann Resonance field strength.
2. High-density EEG with source localization: Extend analysis to high-density recordings to clarify spatial organization of φ^*n*^ generators and validate the frontal hub topology suggested by transfer entropy analysis.
3. Trivariate cross-frequency coupling: Standard bivariate measures (phase-amplitude coupling) may miss critical trivariate interactions predicted by the golden triplet framework (Kramer, 2022). Development of trivariate coupling measures—potentially extending bicoherence analysis to capture three-rhythm resonance (*f*_*k*−1_+*f*_*k*_→*f*_*k*+1_)—could reveal coupling patterns invisible to current methods.
4. Cross-species validation: Test whether φ^*n*^ architecture is evolutionarily conserved across species with different brain sizes. Preliminary evidence suggests similar frequency organization in mammals despite 17,000-fold brain volume variation (Buzsáki et al., 2013).
5. Developmental trajectory: Does φ^*n*^ organization emerge with neural maturation, or is it present from early development?
6. Clinical biomarkers: Do disorders with oscillatory abnormalities (schizophrenia, Parkinson's, ADHD) show disrupted φ^*n*^ organization? Could restoration serve as treatment target?
7. Entrainment studies: Test whether tACS at φ^*n*^ frequencies produces stronger entrainment or superior cognitive effects compared to non-φ^*n*^ frequencies (Herrmann et al., 2016).
8. Behavioral correlates: Examine whether SIE precision or aggregate φ^*n*^ alignment predicts cognitive performance, creativity, or meditative depth.
9. Data-driven boundary validation: Cohen (2021) developed gedBounds, a multivariate method that identifies empirical frequency boundaries through covariance-based clustering. Applying this approach to test whether data-driven boundaries converge on φ^*n*^ integer positions would provide independent validation from a fundamentally different analytical framework. The method also enables examination of individual differences in boundary positioning relative to the population-level φ^*n*^ lattice.
10. Per-band enrichment normalization: The aggregate enrichment analysis reported here pools peaks across all frequency bands. Per-band analysis with band-appropriate spectral resolution and density normalization may reveal band-specific position preferences not captured by the aggregate methodology, and represents a natural extension of the present work.

## 7 Significance statement

Since Hans Berger discovered the alpha rhythm in 1929, EEG frequency bands have been defined by empirical observation rather than theoretical principles. This study provides large-scale empirical validation of the golden ratio (φ = 1.618) organization of neural oscillations theorized by Pletzer et al. (2010), identifying the absolute fundamental frequency *f*_0_ = 7.6 Hz that anchors a φ^*n*^ lattice governing spectral peak distributions. Two independent methodological approaches—transient event detection across 91 participants and single-channel spectral parameterization of over 850,000 oscillatory peaks—converge on identical conclusions: spectral peaks are depleted at φ^*n*^ band boundaries and enriched at band centers. Gamma oscillations show the strongest aggregate adherence, consistent with their stringent requirements for precise temporal binding. These findings support a substrate-ignition model in which the φ^*n*^ lattice exists continuously as an architectural scaffold, with transient high-coherence states representing moments of amplified compliance. The golden ratio's unique mathematical properties—maximal resistance to mode-locking combined with Fibonacci-mediated coupling—may represent an optimal neural solution for balancing segregation and integration across frequency bands.

## 8 Conclusions

This study makes four principal contributions to the understanding of neural oscillatory organization:

First, φ^*n*^ architecture is validated in continuous spectral organization. Systematic analysis of 244,955 spectral peaks—detected agnostically via FOOOF without reference to φ^*n*^ predictions—confirms the predicted boundary-attractor structure in aggregate cross-band analysis: boundaries depleted (−18%), attractors enriched (+21%), noble positions maximally enriched (+39%). Independent replication in 612,990 peaks from the EEGEmotions-27 dataset strengthens this finding. This analysis provides the most robust evidence for φ^*n*^ organization, as it does not depend on event detection pipeline assumptions.

Second, Schumann Ignition Events are established as reproducible transient phenomena. Across 1,366 events, 91 participants, five cognitive contexts, and three consumer EEG devices, SIEs showed consistent spectral, temporal, and network characteristics. The coherence-first temporal signature suggests these represent qualitatively distinct neural states. These findings should be interpreted with awareness that the detection pipeline's search windows partially constrain outcomes, though observed precision exceeds geometric baselines.

Third, the substrate-ignition model is proposed as an integrative framework. The φ^*n*^ lattice exists continuously as an architectural constraint; SIEs represent transient amplification of this substrate. This model explains both the precision of transient events and the robustness of aggregate spectral organization.

Fourth, φ^*n*^ ratio relationships are observed in SIE harmonics that complement the continuous architecture. Analysis of harmonic ratios revealed < 1% deviation from golden ratio predictions. Shuffled bootstrap analysis indicates this precision emerges primarily from population-level distributional properties rather than within-event coordination, consistent with the substrate-ignition model where continuous φ^*n*^ organization is transiently amplified during SIE states.

The core finding can be summarized in a single equation (Equation 28):


$$\begin{array}{l} f\left(n\right)=f_{0}\times \varphi^{n}\text{ where}f_{0}=7.60\text{Hz},\;\varphi =1.6180339... \end{array}$$ (28)

Whether the correspondence between neural *f*_0_ and Earth's Schumann Resonance reflects evolutionary optimization, biophysical convergence, or coincidence cannot be determined without concurrent geomagnetic measurement. What the present findings establish is that human neural oscillations exhibit precise φ^*n*^ ratio organization—a mathematical architecture that may represent a universal solution to the segregation-integration balance required for complex neural computation.

## Data Availability

The analysis code (FOOOF spectral parameterization pipeline, SIE detection, statistical analyses, figure generation) is publicly available at https://github.com/neurokinetikz/research. Validation datasets analyzed in this study were obtained from the following public sources: PhySF (Irshad et al., 2023), MultiPENG (Rashed et al., 2025), VEP (Tiwari et al., 2025), and EEGEmotions-27 (Phuong et al., 2025); access procedures are described in the original publications. The longitudinal meditation EEG recordings used as the discovery dataset are available from the corresponding author upon reasonable request, subject to privacy considerations of single-subject longitudinal data. FOOOF peak detection outputs and derived analysis files are available as supplementary data.
