# Supplementary material for: Schumann-anchored golden ratio organization of human neural oscillations
Source: Front Comput Neurosci. 2026 Jun 2;20:1786996. doi: 10.3389/fncom.2026.1786996 (PMC13269411; doi:10.3389/fncom.2026.1786996)
Supplement: Supplementary file 1 [file Supplementary_file_1.docx]

**Supplemental Tables: Complete** *φ^n^* **Frequency Predictions**

These tables provide comprehensive frequency predictions for all position types within each *φ*-octave band. Frequencies are calculated using *f* (*n*) = 7*.*60 × *φ^n^* where *φ* = 1*.*6180339887. Position types include: boundaries (integer *n*), attractors (*n* + 0*.*5), nobles (1◦ through 7◦ at *n* + *φ*^−^*^k^* for *k* = 1 *. . .* 7), and inverse nobles (1◦ through 7◦ at *n* + 1 − *φ*^−^*^k^*). Note that 2◦ Noble and 1◦ Inverse coincide at offset 0.382, and 1◦ Noble and 2◦ Inverse coincide at offset 0.618, reflecting the fundamental symmetry of the noble hierarchy about the attractor.

Table S1: Sub-delta Band (*n* = −6 to −5, 0.42–0.69 Hz) Position Type Offset *n* Value *φ^n^* Frequency (Hz)

**Boundary** 0.000 −6*.*000 0*.*0557 0.42

7◦ Noble 0.034 −5*.*966 0*.*0566 0.43

6◦ Noble 0.056 −5*.*944 0*.*0573 0.44

5◦ Noble 0.090 −5*.*910 0*.*0582 0.44

4◦ Noble 0.146 −5*.*854 0*.*0598 0.45

3◦ Noble 0.236 −5*.*764 0*.*0624 0.47

2◦ Noble / 1◦ Inverse 0.382 −5*.*618 0*.*0670 0.51

**Attractor** 0.500 −5*.*500 0*.*0709 0.54

*1*◦ *Noble* / 2◦ Inverse 0.618 −5*.*382 0*.*0750 0.57

3◦ Inverse 0.764 −5*.*236 0*.*0805 0.61

4◦ Inverse 0.854 −5*.*146 0*.*0841 0.64

5◦ Inverse 0.910 −5*.*090 0*.*0863 0.66

6◦ Inverse 0.944 −5*.*056 0*.*0878 0.67

7◦ Inverse 0.966 −5*.*034 0*.*0887 0.67

**Upper Boundary** 1.000 −5*.*000 0*.*0902 0.69

Table S2: Sub-delta Band (*n* = −5 to −4, 0.69–1.11 Hz)

| Position Type | Offset | *n* Value | *φn* | Frequency (Hz) |
| --- | --- | --- | --- | --- |
| **Boundary**  7◦ Noble 6◦ Noble 5◦ Noble 4◦ Noble 3◦ Noble  2◦ Noble / 1◦ Inverse  **Attractor**  *1*◦ *Noble* / 2◦ Inverse 3◦ Inverse  4◦ Inverse 5◦ Inverse 6◦ Inverse 7◦ Inverse  **Upper Boundary** | 0.000  0.034  0.056  0.090  0.146  0.236  0.382  0.500  0.618  0.764  0.854  0.910  0.944  0.966  1.000 | −5*.*000  −4*.*966  −4*.*944  −4*.*910  −4*.*854  −4*.*764  −4*.*618  −4*.*500  −4*.*382  −4*.*236  −4*.*146  −4*.*090  −4*.*056  −4*.*034  −4*.*000 | 0*.*0902  0*.*0917  0*.*0926  0*.*0942  0*.*0967  0*.*1010  0*.*1084  0*.*1147  0*.*1214  0*.*1302  0*.*1360  0*.*1397  0*.*1420  0*.*1435  0*.*1459 | 0.69  0.70  0.70  0.72  0.74  0.77  0.82  0.87  0.92  0.99  1.03  1.06  1.08  1.09  1.11 |
| Table S3: Sub-delta Band (*n* = −4 to −3, 1.11–1.79 Hz) | | | | |
| Position Type | Offset | *n* Value | *φn* | Frequency (Hz) |
| **Boundary**  7◦ Noble 6◦ Noble 5◦ Noble 4◦ Noble 3◦ Noble  2◦ Noble / 1◦ Inverse  **Attractor**  *1*◦ *Noble* / 2◦ Inverse 3◦ Inverse  4◦ Inverse 5◦ Inverse 6◦ Inverse 7◦ Inverse  **Upper Boundary** | 0.000  0.034  0.056  0.090  0.146  0.236  0.382  0.500  0.618  0.764  0.854  0.910  0.944  0.966  1.000 | −4*.*000  −3*.*966  −3*.*944  −3*.*910  −3*.*854  −3*.*764  −3*.*618  −3*.*500  −3*.*382  −3*.*236  −3*.*146  −3*.*090  −3*.*056  −3*.*034  −3*.*000 | 0*.*1459  0*.*1483  0*.*1499  0*.*1524  0*.*1565  0*.*1634  0*.*1753  0*.*1856  0*.*1964  0*.*2107  0*.*2201  0*.*2261  0*.*2298  0*.*2322  0*.*2361 | 1.11  1.13  1.14  1.16  1.19  1.24  1.33  1.41  1.49  1.60  1.67  1.72  1.75  1.77  1.79 |

Table S4: Delta Band (*n* = −3 to −2, 1.79–2.90 Hz)

| Position Type | Offset | *n* Value | *φn* | Frequency (Hz) |
| --- | --- | --- | --- | --- |
| **Boundary**  7◦ Noble 6◦ Noble 5◦ Noble 4◦ Noble 3◦ Noble  2◦ Noble / 1◦ Inverse  **Attractor**  *1*◦ *Noble* / 2◦ Inverse 3◦ Inverse  4◦ Inverse 5◦ Inverse 6◦ Inverse 7◦ Inverse  **Upper Boundary** | 0.000  0.034  0.056  0.090  0.146  0.236  0.382  0.500  0.618  0.764  0.854  0.910  0.944  0.966  1.000 | −3*.*000  −2*.*966  −2*.*944  −2*.*910  −2*.*854  −2*.*764  −2*.*618  −2*.*500  −2*.*382  −2*.*236  −2*.*146  −2*.*090  −2*.*056  −2*.*034  −2*.*000 | 0*.*2361  0*.*2400  0*.*2425  0*.*2465  0*.*2532  0*.*2645  0*.*2837  0*.*3003  0*.*3178  0*.*3410  0*.*3561  0*.*3658  0*.*3718  0*.*3758  0*.*3820 | 1.79  1.82  1.84  1.87  1.92  2.01  2.16  2.28  2.42  2.59  2.71  2.78  2.83  2.86  2.90 |
| Table S5: Delta Band (*n* = −2 to −1, 2.90–4.70 Hz) | | | | |
| Position Type | Offset | *n* Value | *φn* | Frequency (Hz) |
| **Boundary**  7◦ Noble 6◦ Noble 5◦ Noble 4◦ Noble 3◦ Noble  2◦ Noble / 1◦ Inverse  **Attractor**  *1*◦ *Noble* / 2◦ Inverse 3◦ Inverse  4◦ Inverse 5◦ Inverse 6◦ Inverse 7◦ Inverse  **Upper Boundary** | 0.000  0.034  0.056  0.090  0.146  0.236  0.382  0.500  0.618  0.764  0.854  0.910  0.944  0.966  1.000 | −2*.*000  −1*.*966  −1*.*944  −1*.*910  −1*.*854  −1*.*764  −1*.*618  −1*.*500  −1*.*382  −1*.*236  −1*.*146  −1*.*090  −1*.*056  −1*.*034  −1*.*000 | 0*.*3820  0*.*3883  0*.*3924  0*.*3989  0*.*4098  0*.*4279  0*.*4590  0*.*4859  0*.*5143  0*.*5517  0*.*5761  0*.*5918  0*.*6016  0*.*6080  0*.*6180 | 2.90  2.95  2.98  3.03  3.11  3.25  3.49  3.69  3.91  4.19  4.38  4.50  4.57  4.62  4.70 |

Table S6: Theta Band (*n* = −1 to 0, 4.70–7.60 Hz)

| Position Type | | Offset | *n* Value | *φn* | Frequency (Hz) |
| --- | --- | --- | --- | --- | --- |
| **Boundary**  7◦ Noble 6◦ Noble 5◦ Noble 4◦ Noble 3◦ Noble  2◦ Noble / 1◦ Inverse  **Attractor**  *1*◦ *Noble* / 2◦ Inverse 3◦ Inverse  4◦ Inverse 5◦ Inverse 6◦ Inverse 7◦ Inverse  **Upper Boundary** | | 0.000  0.034  0.056  0.090  0.146  0.236  0.382  0.500  0.618  0.764  0.854  0.910  0.944  0.966  1.000 | −1*.*000  −0*.*966  −0*.*944  −0*.*910  −0*.*854  −0*.*764  −0*.*618  −0*.*500  −0*.*382  −0*.*236  −0*.*146  −0*.*090  −0*.*056  −0*.*034  0*.*000 | 0*.*6180  0*.*6282  0*.*6349  0*.*6454  0*.*6630  0*.*6924  0*.*7428  0*.*7862  0*.*8321  0*.*8926  0*.*9322  0*.*9576  0*.*9734  0*.*9838  1*.*0000 | 4.70  4.77  4.83  4.90  5.04  5.26  5.64  5.97  6.32  6.78  7.08  7.28  7.40  7.48  7.60 |
|  | Table S7: Alpha Band (*n* = 0 to 1, 7.60–12.30 Hz) | | | | |
| Position Type | | Offset | *n* Value | *φn* | Frequency (Hz) |
| **Boundary** | | 0.000 | 0*.*000 | 1*.*0000 | 7.60 |
| 7◦ | Noble | 0.034 | 0*.*034 | 1*.*0165 | 7.73 |
| 6◦ | Noble | 0.056 | 0*.*056 | 1*.*0273 | 7.81 |
| 5◦ | Noble | 0.090 | 0*.*090 | 1*.*0443 | 7.94 |
| 4◦ | Noble | 0.146 | 0*.*146 | 1*.*0728 | 8.15 |
| 3◦ | Noble | 0.236 | 0*.*236 | 1*.*1203 | 8.51 |
| 2◦ | Noble / 1◦ Inverse | 0.382 | 0*.*382 | 1*.*2018 | 9.13 |
| **Attractor** | | 0.500 | 0*.*500 | 1*.*2720 | 9.67 |
| *1*◦ | *Noble* / 2◦ Inverse | 0.618 | 0*.*618 | 1*.*3463 | 10.23 |
| 3◦ | Inverse | 0.764 | 0*.*764 | 1*.*4443 | 10.98 |
| 4◦ | Inverse | 0.854 | 0*.*854 | 1*.*5083 | 11.46 |
| 5◦ | Inverse | 0.910 | 0*.*910 | 1*.*5495 | 11.78 |
| 6◦ | Inverse | 0.944 | 0*.*944 | 1*.*5750 | 11.97 |
| 7◦ | Inverse | 0.966 | 0*.*966 | 1*.*5918 | 12.10 |
| **Upper Boundary** | | 1.000 | 1*.*000 | 1*.*6180 | 12.30 |

Table S8: Low *β* Band (*n* = 1 to 2, 12.30–19.90 Hz)

| Position Type | | Offset | *n* Value | *φn* | Frequency (Hz) |
| --- | --- | --- | --- | --- | --- |
| **Boundary** | | 0.000 | 1*.*000 | 1*.*6180 | 12.30 |
| 7◦ | Noble | 0.034 | 1*.*034 | 1*.*6447 | 12.50 |
| 6◦ | Noble | 0.056 | 1*.*056 | 1*.*6622 | 12.63 |
| 5◦ | Noble | 0.090 | 1*.*090 | 1*.*6896 | 12.84 |
| 4◦ | Noble | 0.146 | 1*.*146 | 1*.*7358 | 13.19 |
| 3◦ | Noble | 0.236 | 1*.*236 | 1*.*8126 | 13.78 |
| 2◦ | Noble / 1◦ Inverse | 0.382 | 1*.*382 | 1*.*9446 | 14.78 |
| **Attractor** | | 0.500 | 1*.*500 | 2*.*0582 | 15.64 |
| *1*◦ | *Noble* / 2◦ Inverse | 0.618 | 1*.*618 | 2*.*1784 | 16.56 |
| 3◦ | Inverse | 0.764 | 1*.*764 | 2*.*3370 | 17.76 |
| 4◦ | Inverse | 0.854 | 1*.*854 | 2*.*4404 | 18.55 |
| 5◦ | Inverse | 0.910 | 1*.*910 | 2*.*5071 | 19.05 |
| 6◦ | Inverse | 0.944 | 1*.*944 | 2*.*5484 | 19.37 |
| 7◦ | Inverse | 0.966 | 1*.*966 | 2*.*5755 | 19.57 |
| **Upper Boundary** | | 1.000 | 2*.*000 | 2*.*6180 | 19.90 |
|  | Table S9: High *β* Band (*n* = 2 to 3, 19.90–32.19 Hz) | | | | |
| Position Type | | Offset | *n* Value | *φn* | Frequency (Hz) |
| **Boundary** | | 0.000 | 2*.*000 | 2*.*6180 | 19.90 |
| 7◦ | Noble | 0.034 | 2*.*034 | 2*.*6612 | 20.23 |
| 6◦ | Noble | 0.056 | 2*.*056 | 2*.*6895 | 20.44 |
| 5◦ | Noble | 0.090 | 2*.*090 | 2*.*7339 | 20.78 |
| 4◦ | Noble | 0.146 | 2*.*146 | 2*.*8086 | 21.35 |
| 3◦ | Noble | 0.236 | 2*.*236 | 2*.*9329 | 22.29 |
| 2◦ | Noble / 1◦ Inverse | 0.382 | 2*.*382 | 3*.*1464 | 23.91 |
| **Attractor** | | 0.500 | 2*.*500 | 3*.*3302 | 25.31 |
| *1*◦ | *Noble* / 2◦ Inverse | 0.618 | 2*.*618 | 3*.*5248 | 26.79 |
| 3◦ | Inverse | 0.764 | 2*.*764 | 3*.*7813 | 28.74 |
| 4◦ | Inverse | 0.854 | 2*.*854 | 3*.*9487 | 30.01 |
| 5◦ | Inverse | 0.910 | 2*.*910 | 4*.*0565 | 30.83 |
| 6◦ | Inverse | 0.944 | 2*.*944 | 4*.*1234 | 31.34 |
| 7◦ | Inverse | 0.966 | 2*.*966 | 4*.*1673 | 31.67 |
| **Upper Boundary** | | 1.000 | 3*.*000 | 4*.*2361 | 32.19 |

Table S10: *γ* Band (*n* = 3 to 4, 32.19–52.09 Hz)

| Position Type | | Offset | *n* Value | *φn* | Frequency (Hz) |
| --- | --- | --- | --- | --- | --- |
| **Boundary** | | 0.000 | 3*.*000 | 4*.*2361 | 32.19 |
| 7◦ | Noble | 0.034 | 3*.*034 | 4*.*3059 | 32.73 |
| 6◦ | Noble | 0.056 | 3*.*056 | 4*.*3518 | 33.07 |
| 5◦ | Noble | 0.090 | 3*.*090 | 4*.*4236 | 33.62 |
| 4◦ | Noble | 0.146 | 3*.*146 | 4*.*5444 | 34.54 |
| 3◦ | Noble | 0.236 | 3*.*236 | 4*.*7455 | 36.07 |
| 2◦ | Noble / 1◦ Inverse | 0.382 | 3*.*382 | 5*.*0909 | 38.69 |
| **Attractor** | | 0.500 | 3*.*500 | 5*.*3884 | 40.95 |
| *1*◦ | *Noble* / 2◦ Inverse | 0.618 | 3*.*618 | 5*.*7032 | 43.34 |
| 3◦ | Inverse | 0.764 | 3*.*764 | 6*.*1183 | 46.50 |
| 4◦ | Inverse | 0.854 | 3*.*854 | 6*.*3891 | 48.56 |
| 5◦ | Inverse | 0.910 | 3*.*910 | 6*.*5636 | 49.88 |
| 6◦ | Inverse | 0.944 | 3*.*944 | 6*.*6719 | 50.71 |
| 7◦ | Inverse | 0.966 | 3*.*966 | 6*.*7429 | 51.25 |
| **Upper Boundary** | | 1.000 | 4*.*000 | 6*.*8541 | 52.09 |
